# Targeting KAT2A inhibits inflammatory macrophage activation and rheumatoid arthritis through epigenetic and metabolic reprogramming

**DOI:** 10.1002/mco2.306

**Published:** 2023-06-11

**Authors:** Yunkai Zhang, Ying Gao, Yingying Ding, Yuyu Jiang, Huiying Chen, Zhenzhen Zhan, Xingguang Liu

**Affiliations:** ^1^ Department of Pathogen Biology Naval Medical University Shanghai China; ^2^ National Key Laboratory of Immunity & Inflammation Naval Medical University Shanghai China; ^3^ Department of Rheumatology Changhai Hospital, Naval Medical University Shanghai China; ^4^ Key Laboratory of Arrhythmias of the Ministry of Education of China Shanghai East Hospital, Tongji University School of Medicine Shanghai China; ^5^ Department of Liver Surgery, Shanghai Institute of Transplantation Renji Hospital, Shanghai Jiao Tong University School of Medicine Shanghai China

**Keywords:** KAT2A, metabolic reprogramming, NLRP3, NRF2, rheumatoid arthritis

## Abstract

Epigenetic regulation of inflammatory macrophages governs inflammation initiation and resolution in the pathogenesis of rheumatoid arthritis (RA). Nevertheless, the mechanisms underlying macrophage‐mediated arthritis injuries remain largely obscure. Here, we found that increased expression of lysine acetyltransferase 2A (KAT2A) in synovial tissues was closely correlated with inflammatory joint immunopathology in both RA patients and experimental arthritis mice. Administration of MB‐3, the KAT2A‐specific chemical inhibitor, significantly ameliorated the synovitis and bone destruction in collagen‐induced arthritis model. Both pharmacological inhibition and siRNA silencing of KAT2A, not only suppressed innate stimuli‐triggered proinflammatory gene (such as *Il1b* and *Nlrp3*) transcription but also impaired NLR family pyrin domain containing 3 (NLRP3) inflammasome activation in vivo and in vitro. Mechanistically, KAT2A facilitated macrophage glycolysis reprogramming through suppressing nuclear factor‐erythroid 2‐related factor 2 (NRF2) activity as well as downstream antioxidant molecules, which supported histone 3 lysine 9 acetylation (H3K9ac) and limited NRF2‐mediated transcriptional repression of proinflammatory genes. Our study proves that acetyltransferase KAT2A licenses metabolic and epigenetic reprogramming for NLRP3 inflammasome activation in inflammatory macrophages, thereby targeting KAT2A represents a potential therapeutic approach for patients suffering from RA and relevant inflammatory diseases.

## Graphical abstract

KAT2A licenses the epigenetic and metabolic reprogramming of inflammatory macrophages. In synovial tissues of RA patients, the increased expression of KAT2A promotes the inducible transcription of *Il1b* and *Nlrp3* genes by catalyzing H3K9ac and limiting NRF2 activation, thereby leading to uncontrolled IL‐1β production and inflammatory injury in RA progression.

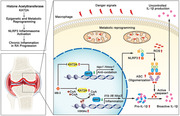

## 1 INTRODUCTION

Rheumatoid arthritis (RA) is one of the most prevalent chronic inflammatory and autoimmune diseases characterized by synovial inflammation as well as hyperplasia. The pathogenesis of RA involves many kinds of innate and adaptive immune cells, including macrophages, neutrophils, autoimmune inflammatory T helper type 1 (Th1) cells, and Th17 cells, which are rapidly infiltrated into the inflamed joint after activation.^1^, ^2^ Pathogenic macrophages and T cells interact with tissue‐resident fibroblast‐like synoviocytes for destroying lesioned cartilage and bone. It was well accepted that macrophages bridge the innate and adaptive immune responses when they present the self‐antigens to T cells and secrete proinflammatory cytokines, such as interleukin‐1β (IL‐1β) and tumor necrosis factor‐α (TNF‐α), in the development and maintenance of chronic inflammatory disorders.^3^, ^4^ Nevertheless, many researchers were devoted to the novel effective therapy for RA patients, and these targeting inflammatory macrophages for immune regulation show tremendous therapeutic potential.

Epigenetic modifications are widely acknowledged as hereditable transcriptional regulation of gene expression without altering genome sequence and play an important part in many biological and pathological processes.^5^ Increasing evidence shows that the proinflammatory transition of macrophages is critically controlled by several epigenetic modifications, including histone modification, DNA methylation, chromatin remodeling, and so on. For example, lysine methyltransferase 2B epigenetically regulated the key enzyme expression in glycosylphosphatidylinositol anchor synthesis by catalyzing histone H3 lysine 4 tri‐methylation (H3K4me3) at gene promoter, which led to the control of toll‐like receptor (TLR)‐mediated effector macrophage function.^6^ Additionally, ASH1 like histone lysine methyltransferase promoted the expression of ubiquitin‐editing enzyme A20 by modulating H3K4me3 level to suppress nuclear factor kappa B (NF‐κB) signaling and myeloid cell responses in sepsis and experimental arthritis.^7^ In this way, understanding the epigenetic mechanisms of macrophage function will broaden our horizon of prevention and treatment of inflammatory and autoimmune diseases.

Histone acetylation is catalyzed by several classical histone acetyltransferase (HAT), which adds acetyl group to lysine on histone tails. Aberrant histone acetylation is tightly associated with the transcription disorder and pathogenic gene expression, which has been proved to be potential diagnostic biomarkers or therapeutic targets for immune diseases.^8^, ^9^ For example, histone deacetylation in oligodendrocytes manifested early‐stage demyelination in central nervous system lesions of multiple sclerosis patients.^10^ Our previous work demonstrated that lysine acetyltransferase KAT8 suppressed type I interferon (IFN‐I) production via direct acetylation of lysine 359 of interferon regulatory factor 3, which might be a promising intervention for treating IFN‐I‐associated inflammatory diseases such as lupus and psoriasis.^11^ However, the functions of other host factors in macrophages participated in histone and nonhistone acetylation for inflammation initiation and resolution remain largely unknown.

Lysine acetyltransferase 2A (KAT2A), also named as general control nondepressible 5 (GCN5), was initially identified as the important member of HAT family in yeast and turned out to be highly conserved in mammals, especially in humans and mice.^12^ Germline KAT2A deletion in mice resulted in embryonic lethality with mesodermal defects.^13^ Increasing studies showed that KAT2A functioned as a critical regulator in various physiological and pathological processes, such as gene regulation, cellular metabolism, and neurological diseases.^14^ For example, KAT2A facilitated the G2/M progression and cell proliferation through directly promoting α‐tubulin acetylation.^15^ KAT2A catalyzed the increased H3K9ac levels of the *VEGF* promoter for bone marrow‐derived mesenchymal cell‐mediated angiogenesis.^16^ However, the role of KAT2A in immune system, especially in macrophage‐mediated NLRP3 (NLR family pyrin domain containing 3) inflammasome activation and inflammatory disease is still largely unknown. Here, we found that administration of a selective KAT2A inhibitor, MB‐3, markedly ameliorated the inflammatory injure and bone destruction in collagen‐induced arthritis (CIA) model. KAT2A silencing or inhibitor not only suppressed innate stimuli‐triggered *Il1b* and *Nlrp3* transcription but also impaired NLRP3 inflammasome assembly through modulating nuclear factor‐erythroid 2‐related factor 2 (NRF2) activation and inhibiting the glycolysis reprogramming in macrophages. Our data reveal the novel therapeutic target for KAT2A in metabolic and epigenetic reprogramming of macrophages to regulate inflammatory responses in the initiation and progress of RA.

## 2 RESULTS

### 2.1 Increased KAT2A expression is correlated with joint inflammation in both human and mice

**FIGURE 1:**
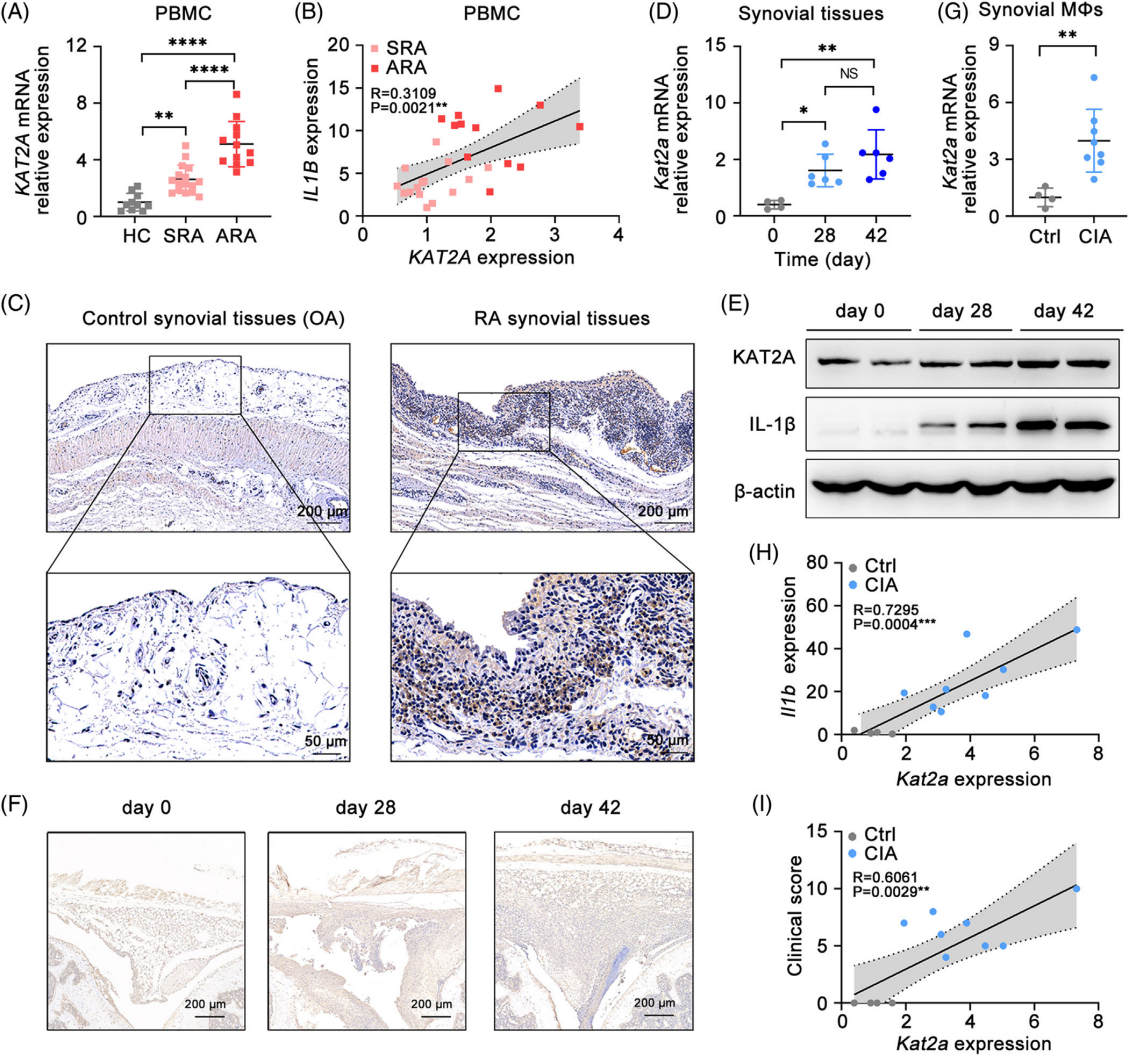
Increased expression of KAT2A is significantly correlated with joint inflammation in both RA patients and CIA model mice. (A) Q‐PCR analysis of *KAT2A* mRNA level in PBMCs from healthy controls (HCs, *n* = 10), stable rheumatoid arthritis patients (SRA, *n* = 16), and active rheumatoid arthritis patients (ARA, *n* = 12). (B) Correlation analysis of *KAT2A* mRNA level with *IL1B* mRNA level in PBMCs from SRA and ARA patients (*n* = 28). (C) IHC staining of KAT2A in paraffin‐embedded sections of synovial tissues from OA and RA patients. Scale bar: 200 μm (upper) or 50 μm (lower). (D and E) Q‐PCR analysis of *Kat2a* mRNA level (*n* = 6 mice per group) (D) and KAT2A protein level (*n* = 2 mice per group) (E) in synovial tissues from CIA model mice at the indicated times. (F) IHC staining of KAT2A in paraffin‐embedded sections of synovial tissues from CIA model mice. Scale bar: 200 μm. (G) Q‐PCR analysis of *Kat2a* mRNA level in synovial macrophages from CIA model mice at day 28 after the first immunization (n = 4−8 mice per group). (H and I) Correlation analysis of *Kat2a* mRNA level with *Il1b* mRNA level (H) in synovial macrophages and clinical score (I) of CIA model mice at day 28 after the first immunization. ^*^ *p* < 0.05; ^**^ *p* < 0.01; ^***^ *p* < 0.001, ^****^ *p* < 0.0001. One‐way ANOVA (A and D), unpaired Student's *t*‐test (G), simple linear regression (B, H, and I).

Considering the potential function of KAT2A in the pathogenesis of RA, we collected the peripheral blood mononuclear cell (PBMC) samples from health controls, stable RA (SRA) patients, active RA (ARA) patients, who fulfilled the diagnostic criteria given by American College of Rheumatology,^17^ and analyzed *KAT2A* mRNA expression. As expected, *KAT2A* mRNA levels in RA patients, especially in ARA patients, were significantly higher than that in health controls (Figure 1A). Interestingly, RA patients with higher *KAT2A* mRNA levels also had correspondingly higher expression levels of *IL1B* (Figure 1B), which has been proven as the key pathogenic factor in RA.^18^ Further analysis of KAT2A expression in synovial tissues from osteoarthritis (OA) and RA samples by immunohistochemical (IHC) directly confirmed the increased KAT2A expression in inflamed knee joints from RA patients (Figure 1C).

Next, we performed the CIA model, a well‐established animal model with pathological characteristic similar to RA patients,^19^ to assess the change of KAT2A expression in mouse synovial tissues. With the progress of arthritis, both mRNA and protein expression of KAT2A in synovial tissue samples of CIA model was remarkably increased, consistent with the phenomenon in human RA patients (Figures 1D–F). Next, we detected KAT2A expression in synovial F4/80^+^ macrophages, the major immune cells that produce pathogenic IL‐1β in joint cavity. *Kat2a* mRNA expression in synovial macrophages from CIA model was obviously higher after arthritis induction (Figure 1G). Furthermore, *Kat2a* expression was strongly correlated with *Il1b* expression in synovial macrophages and clinical score of arthritis mice (Figures 1H and I). These data indicate that KAT2A expression is positively correlated with the joint inflammation in both RA patient and mouse CIA model.

### 2.2 KAT2A inhibitor ameliorates the inflammatory pathology of arthritis

**FIGURE 2:**
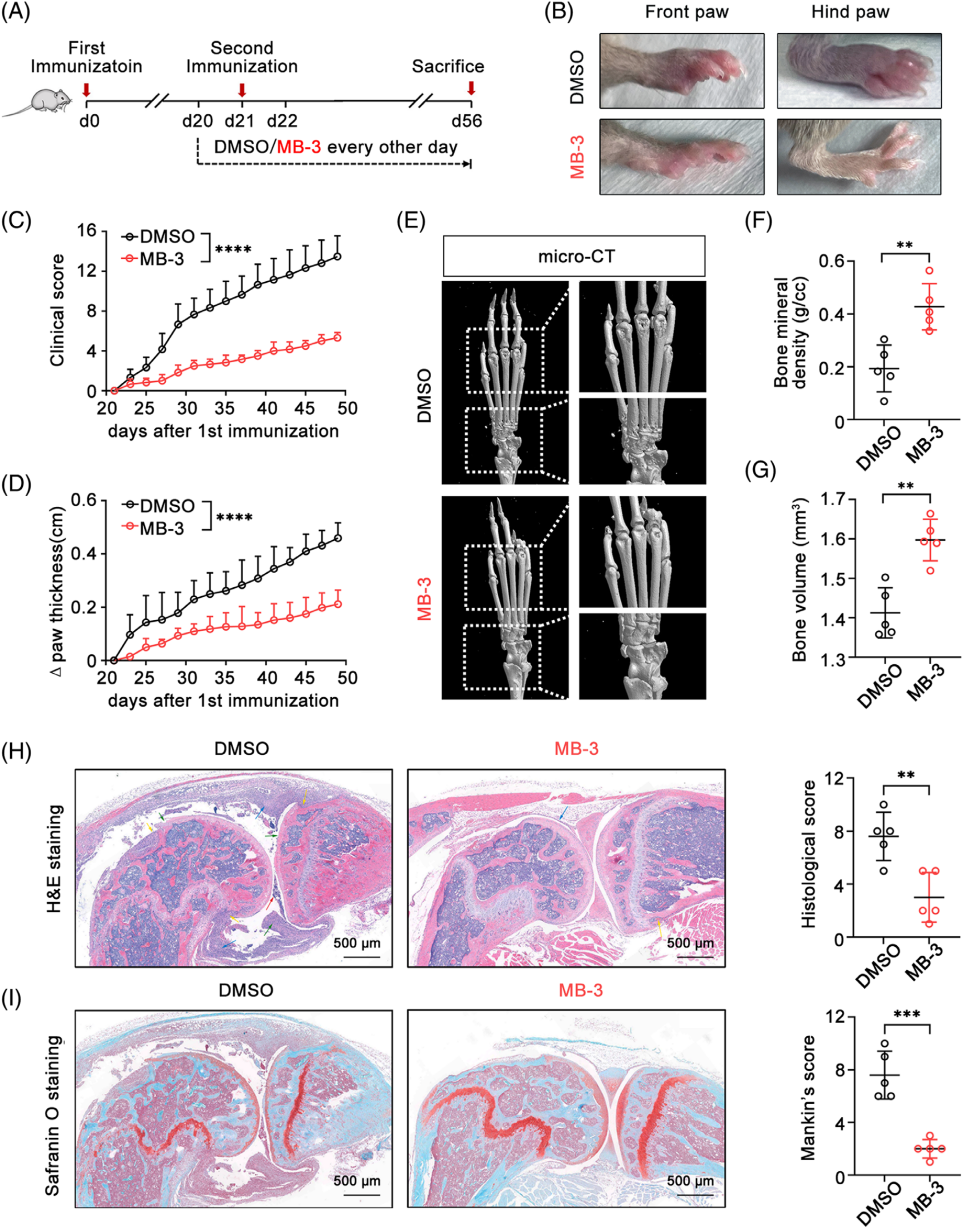
KAT2A inhibitor alleviates the inflammatory pathology and tissue damage in CIA model mice. (A) Experimental design for MB‐3 (10 mg/kg body weight, i.p.) or DMSO administration in CIA model mice. (B–D) Morphology of the indicated paws (B), clinical scores (C), and the increased thickness of hind paws (D) from CIA model mice administrated with MB‐3 or DMSO as in A (*n* = 6 mice per group). (E–G) Micro‐CT images (E), bone mineral density (F), and bone volume (G) of hind paws from CIA model mice administrated with MB‐3 or DMSO as in A (*n* = 5 mice per group). (H and I) H&E staining and histological scores (H), Safranin O staining and Mankin's scores (I) of knee joints from CIA model mice administrated with MB‐3 or DMSO as in A (*n* = 5 mice per group). Scale bar: 500 μm. ^**^ *p* < 0.01; ^***^ *p* < 0.001, ^****^ *p* < 0.0001. Two‐way ANOVA (C and D), unpaired Student's *t*‐test (F, G, H, and I).

To explore the role of KAT2A in the pathological process of RA, CIA model mice were administrated with a selective KAT2A enzymatic inhibitor MB‐3^20^, ^21^ (Figure 2A). MB‐3 administration remarkably inhibited the development of inflammatory arthritis, with lower clinical score and much milder joint swelling in MB‐3‐treated mice (Figures 2B–D). Three‐dimensional micro‐computed tomography (micro‐CT) revealed that MB‐3 administration alleviated the extent of bone erosion in finger phalanges and wrist joints, thus improving bone density and bone volume (Figures 2E–G). Furthermore, histological analysis of knee joints showed the less joint capsule swelling, attenuated synovial hyperplasia, and reduced inflammatory cell infiltration in knee joints of CIA model mice with MB‐3 administration (Figure 2H). Safranin O staining found that KAT2A inhibition also suppressed cartilage destruction of knee joints in CIA mice (Figure 2I). These above data demonstrate that KAT2A inhibition significantly alleviates the pathological damage of arthritis, implying that KAT2A is a key pathogenic factor for arthritis.

### 2.3 KAT2A inhibitor suppresses inflammation and immune disorder in CIA model mice

**FIGURE 3:**
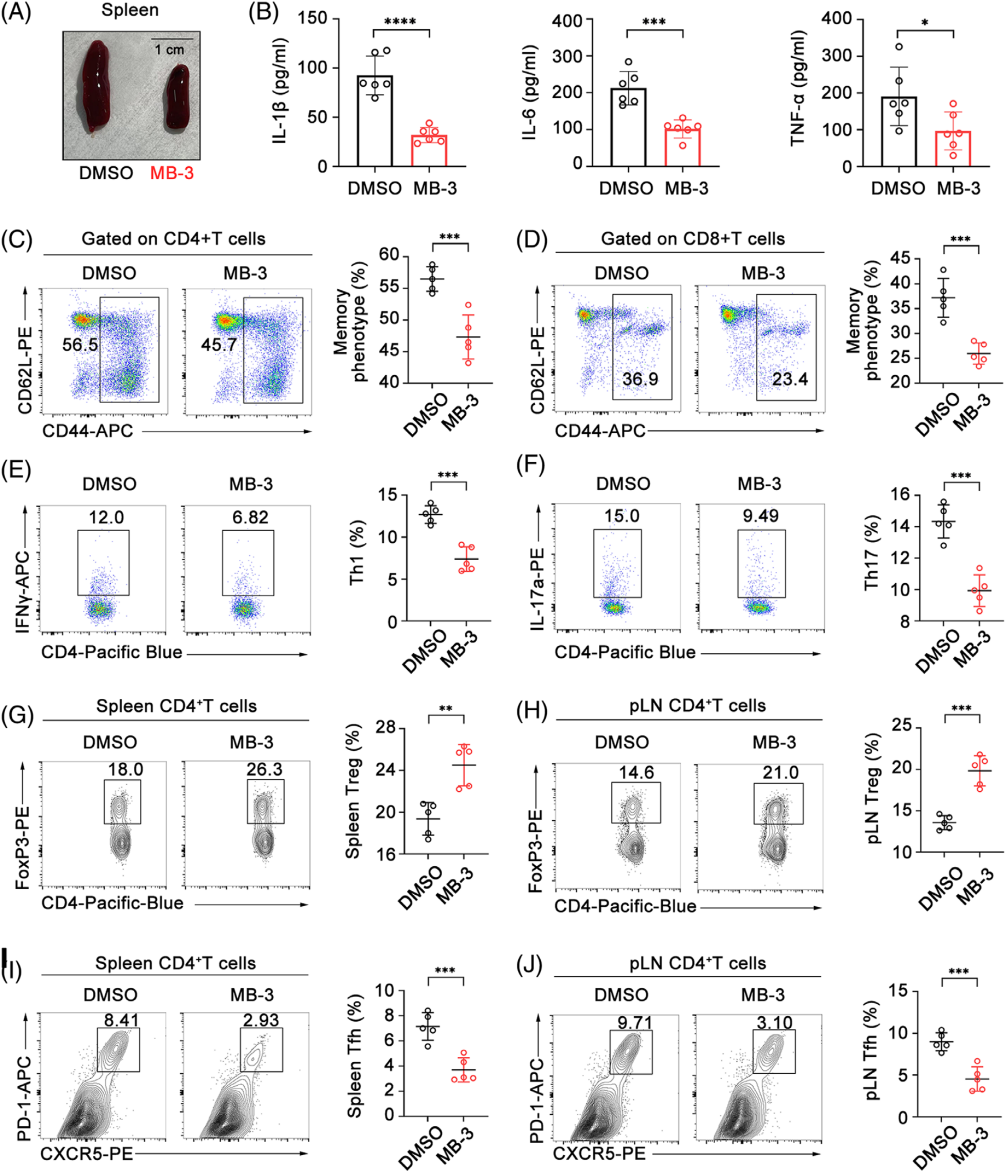
KAT2A inhibitor ameliorates inflammation and immune disorder in CIA model mice. (A) Morphology of spleens from CIA model mice administrated with MB‐3 (10 mg/kg body weight, i.p.) or DMSO. (B) ELISA analysis of concentration of the indicated cytokines in the sera from CIA model mice administrated with MB‐3 or DMSO (*n* = 6 mice per group). (C and D) Flow cytometric analysis of the percentages of CD44^+^ memory phenotype CD4^+^ T cells (C) and CD44^+^ memory phenotype CD8^+^ T cells (D) in spleens from CIA model mice administrated with MB‐3 or DMSO (*n* = 5 mice per group). (E and F) Flow cytometric analysis of the percentages of IFN‐γ‐producing CD4^+^ T cells (E) and IL‐17a‐producing CD4^+^ T cells (F) in spleens from CIA model mice administrated with MB‐3 or DMSO (*n* = 5 mice per group). (G and H) Flow cytometric analysis of the percentages of Treg cells in spleens (G) and pLNs (H) from CIA model mice administrated with MB‐3 or DMSO (*n* = 5 mice per group). (I and J) Flow cytometric analysis of the percentages of Tfh cells in spleens (I) and pLNs (J) from CIA model mice administrated with MB‐3 or DMSO (*n* = 5 mice per group). ^*^ *p* < 0.05; ^**^ *p* < 0.01; ^***^ *p* < 0.001; ^****^ *p* < 0.001. Unpaired Student's *t*‐test (B–J).

Immune dysregulation largely determines RA pathogenesis.^4^ Notably, we observed that pathological splenomegaly in control CIA mice was improved by MB‐3 administration, implying that KAT2A inhibition might directly influence the inflammatory pathology in CIA model (Figure 3A). Then, we analyzed the secretion of proinflammatory cytokines in sera. As expected, there was a significant reduction of IL‐1β, IL‐6, and TNF‐α concentration in sera of CIA mice administrated with MB‐3 (Figure 3B). Previous studies showed that these proinflammatory cytokines were important in T cell proliferation and polarization, which contributed to the progress of RA.^22^, ^23^ Flow cytometric analysis revealed the reduction in both CD4^+^ and CD8^+^ T cell population that highly expressed CD44, a marker of murine memory T cells from MB‐3‐treated CIA mice (Figures 3C and D). The imbalance between proinflammatory and anti‐inflammatory T cells populations dysregulated in RA leading to severe clinical outcomes.^24^, ^25^ Thereby, we analyzed the populations of Th1 and Th17 that produced interferon‐γ (IFN‐γ) and IL‐17a respectively in CIA model mice, and found that MB‐3 administration greatly reduced the frequency of both Th1 and Th17 cells (Figures 3E and F). By contrast, increased frequency of regulatory T cells (Tregs) was found in both spleens and popliteal lymph nodes (pLNs) from MB‐3‐treated mice (Figures 3G and H). Follicular helper T (Tfh) cells are one subgroup of activated CD4^+^ T cells that contribute to the formation and maintenance of germinal center and aggravate RA immunopathology.^26^ KAT2A inhibition strikingly reduced the population of CD4^+^CXCR5^+^PD‐1^+^ Tfh cells in both spleens and pLNs of CIA model mice (Figures 3I and J). To sum up, KAT2A inhibition ameliorates the immunological injury in RA through inhibiting proinflammatory cytokine production and redressing subsequent T lymphocyte imbalance.

### 2.4 KAT2A inhibitor controls lipopolysaccharide (LPS)‐induced systemic inflammation in vivo

To further investigate the biological function of KAT2A in vivo, we performed the adaptive immune cell (e.g., T and B cells)‐independent acute systemic inflammation model via intraperitoneal (i.p.) injection of LPS, which triggers the activation of innate immune systems, especially macrophages, including production of proinflammatory cytokines as well as infiltration of inflammatory cells.^27^ MB‐3 pretreatment ameliorated the inflammatory injury in lung tissues of LPS‐induced endotoxin shock model with improved pathological changes including the reduced inflammatory cell infiltration and bleeding (Figures S1A and B). As expected, MB‐3 pretreatment led to a great decrease in the serum cytokine concentration including IL‐1β, IL‐6, and TNF‐α in endotoxin shock model (Figure S1C).

Additionally, the i.p. injection of LPS triggers the peritonitis model that is driven by inflammatory macrophages and its secretion of proinflammatory cytokines. We analyzed the peritoneal cell population by flow cytometry and found that MB‐3 pretreatment inhibited the infiltration of F4/80^+^CD11b^+^ macrophages into the peritoneal cavity of mice administrated with LPS (Figures S2A and B). Then, we isolated peritoneal F4/80^+^ macrophages from peritoneal lavage fluids for detection the mRNA levels of proinflammatory cytokines and found a remarkable decrease in mRNA levels of IL‐1β and IL‐6, with no influence on the mRNA level of TNF‐α (Figure S2C). These data indicate that KAT2A inhibition by MB‐3 restricts LPS‐induced systemic inflammation in vivo through modulating the effector function of innate immune cells.

### 2.5 KAT2A is required for NLRP3 inflammasome priming

**FIGURE 4:**
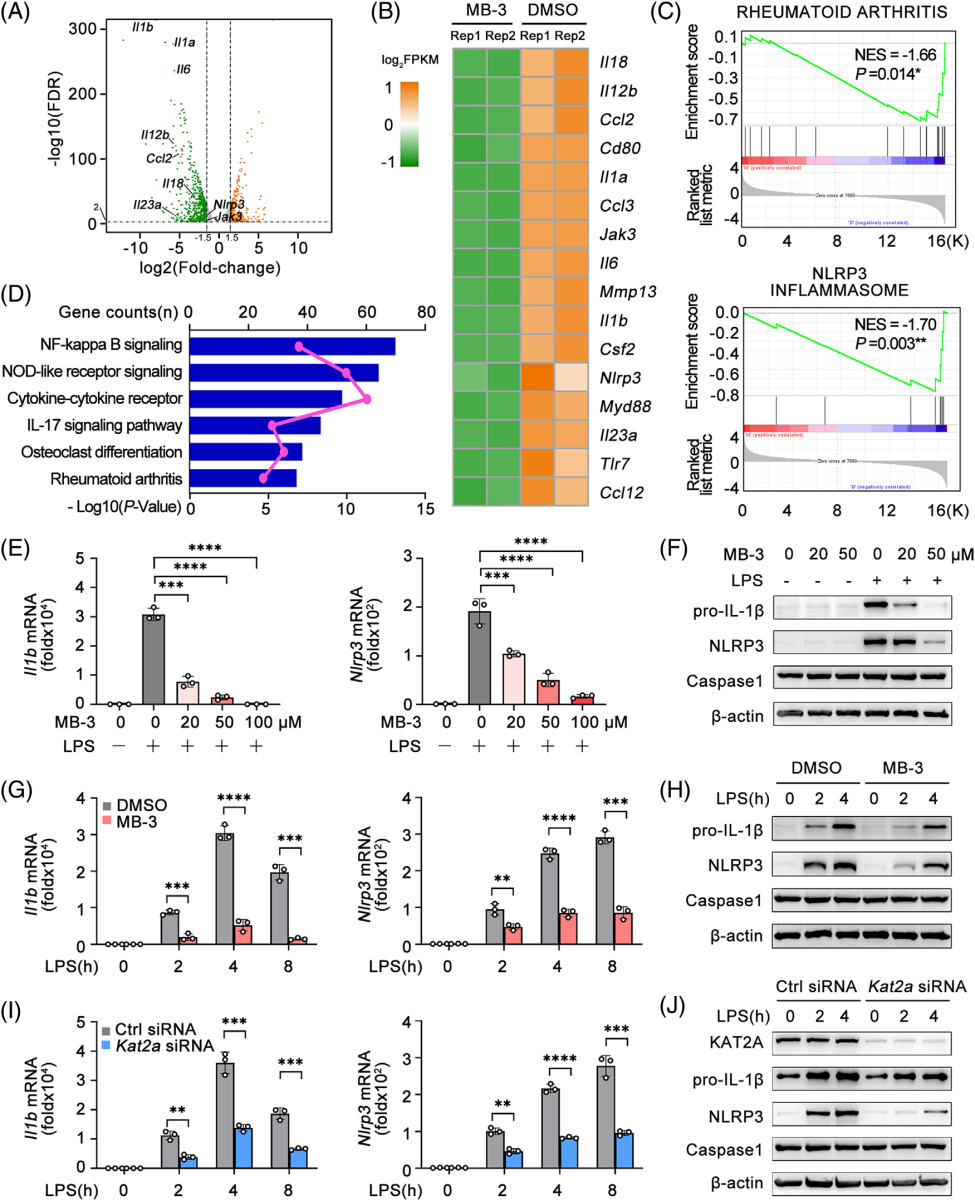
KAT2A is required for NLRP3 inflammasome activation at the priming stage. (A) Scatter plots of gene expression in mouse BMDMs pretreated with MB‐3 (50 μM) or DMSO followed by stimulation with LPS (100 ng/mL) for 4 h. Orange: upregulated genes. Green: downregulated genes. (B–D) Heatmap of differentially expressed gene involving inflammatory responses (B), GSEA analysis (C), and KEGG pathway analysis (D) of BMDMs as in A. Actual numbers of differentially expressed genes were shown as pink lines in D. (E and F) Q‐PCR analysis of the indicated genes (E) and immunoblot analysis of the indicated proteins (F) in BMDMs pretreated with the indicated amounts of MB‐3 followed by stimulation with LPS (100 ng/mL) for 4 h. (G and H) Q‐PCR analysis of the indicated genes (G) and immunoblot analysis of the indicated proteins (H) in BMDMs pretreated with MB‐3 (50 μM) followed by stimulation with LPS (100 ng/mL) for the indicated times. (I and J) Q‐PCR analysis of the indicated genes (I) and immunoblot analysis of the indicated proteins (J) in BMDMs transfected with control siRNA or *Kat2a* siRNA followed by stimulation with LPS (100 ng/mL) for the indicated times. ^**^ *p* < 0.01; ^***^ *p* < 0.001; ^****^ *p* < 0.001. Unpaired Student's *t*‐test (E, G, and I).

In order to investigate the direct role of KAT2A in the macrophage‐mediated inflammatory response, the global gene expression profile induced by LPS in mouse bone marrow‐derived macrophages (BMDMs) with DMSO or MB‐3 treatment was analyzed by the RNA‐sequencing (RNA‐seq). KAT2A inhibition resulted in the giant reduction of several proinflammatory gene, such as *Il1a*, *Il1b*, *Il6*, *Ccl2*, and *Nlrp3* (Figures 4A and B). Compared with DMSO‐treated BMDMs, MB‐3 treatment led to the marked expression difference in genes functioned in RA, consistent with our results revealed by in vivo CIA model (Figure 4C). Notably, the differentially expressed gene mediated by MB‐3 treatment were also enriched in NLRP3 inflammasome pathway (Figure 4C). KEGG pathway showed that upregulated genes were enriched in NF‐κB signaling, NOD‐like receptor signaling, cytokine–cytokine receptor, and RA (Figure 4D).

Increasing evidence has showed that NLRP3 inflammasome makes great contribution to the pathogenesis of RA, and deficiency of genes encoding NLRP3 inflammasome complex proteins exhibits protective function in mouse CIA model.^28^, ^29^ The activation mechanism of NLRP3 inflammasome is well acknowledged: the first step involving the inducible protein expression of pro‐IL‐1β and NLRP3 for priming, and the second one involving the assembly of NLRP3 inflammasome components such as caspase 1, ASC (apoptosis‐associated speck‐like protein), and so on.^30^ In this way, we speculated whether KAT2A played a role in NLRP3 gene expression in inflammatory macrophages and arthritis. We reanalyzed the gene expression in PBMCs from RA patients, and found the strong positive correlation between *KAT2A* and *NLRP3* mRNA expression (Figure S3A). The mRNA expression of *Kat2a* in synovial macrophages from CIA model was also significantly higher after arthritis induction, which was strongly correlated with *Nlrp3* expression in synovial macrophages (Figures S3B and C). Next, we verified the effects of MB‐3 on LPS‐induced *Il1b* and *Nlrp3* transcription in macrophages in vitro. MB‐3 treatment inhibited LPS‐induced gene transcription of IL‐1β and NLRP3 in a dose‐dependent manner (Figure 4E), which was also confirmed by the immunoblot analysis (Figure 4F). Meanwhile, MB‐3 treatment also suppressed the mRNA and protein expression levels of IL‐1β and NLRP3 with different times of LPS stimulation (Figures 4G and H). In addition, we found MB‐3 had the inhibitory role in IL‐6 transcription and secretion, which also proved the results of RNA‐seq (Figures S4A–D). Furthermore, *Kat2a* silencing with the specific siRNA markedly inhibited the inducible expression of IL‐1β and NLRP3 (Figures 4I and J). To investigate the functions of KAT2A thoroughly, we further utilized another two KAT2A‐specific inhibitors CPTH2^31^ and PU139^32^, ^33^ to observe their effects on macrophage activation. The treatment of these two inhibitors both inhibited LPS‐induced transcription of *Il1b* and *Nlrp3* in BMDMs, which was also confirmed by immunoblot assays (Figures S5A–F). In this way, we concluded that pharmacological inhibition of KAT2A by CPTH2 or PU139 also inhibits NLRP3 inflammasome priming. These data indicate KAT2A promotes inducible expression of *Il1b* and *Nlrp3* by LPS stimulation, which is indispensable for NLRP3 inflammasome priming in vitro.

### 2.6 KAT2A promotes NLRP3 inflammasome‐dependent IL‐1β processing

**FIGURE 5:**
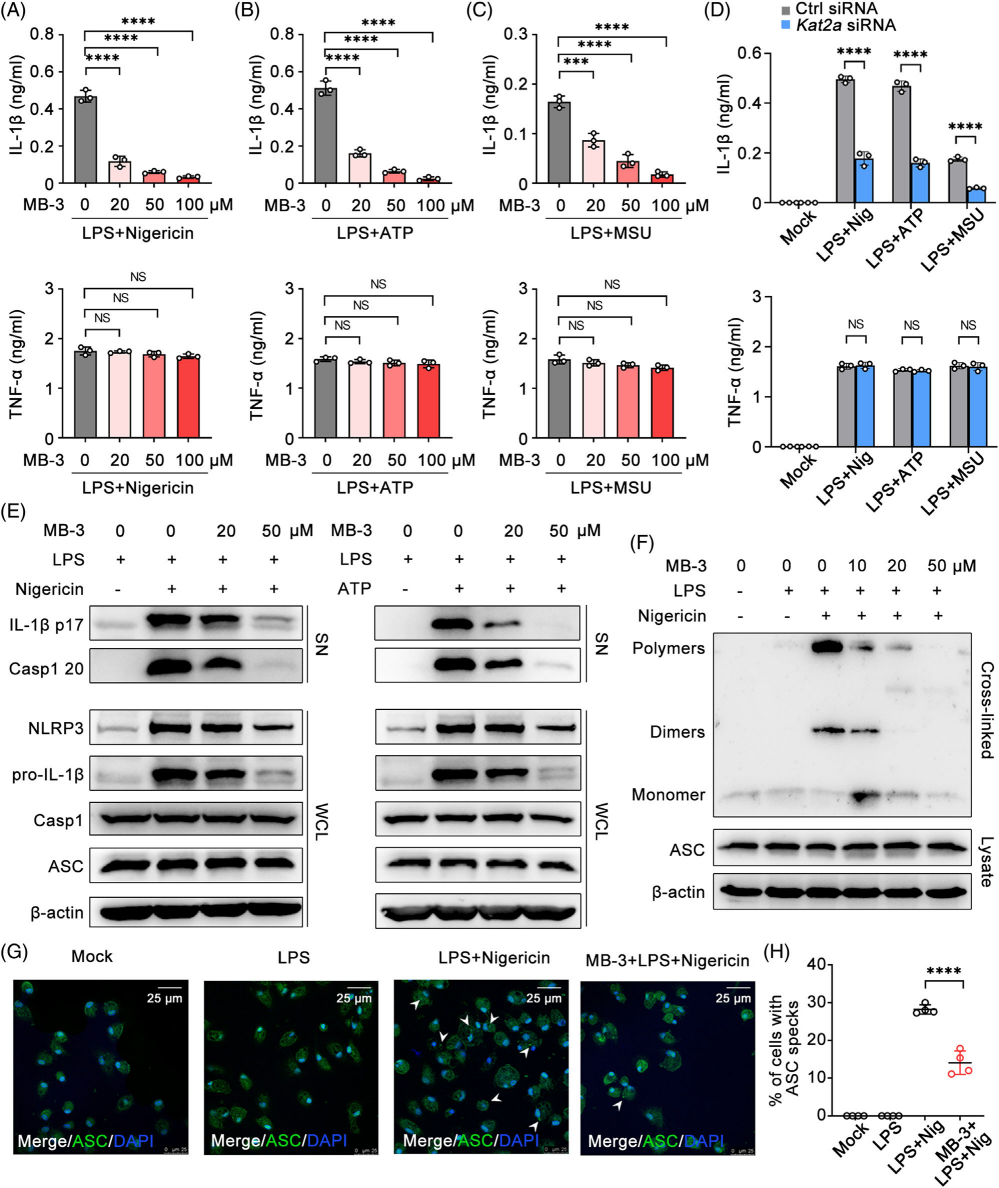
KAT2A promotes NLRP3 inflammasome assembly and IL‐1β processing. (A–C) ELISA analysis of concentration of the indicated cytokines in supernatants of LPS‐primed (100 ng/mL) mouse BMDMs pretreated with MB‐3 followed by stimulation with Nigericin (10 μM) (A), ATP (5 mM) (B), and MSU (200 μg/mL) (C). (D) ELISA analysis of concentration of the indicated cytokines in supernatants of BMDMs transfected with control siRNA or *Kat2a* siRNA followed by treatment as indicated. (E) Immunoblot analysis of proteins from supernatant (SN) and whole cell lysis (WCL) of LPS‐primed (100 ng/mL) BMDMs pretreated with MB‐3 followed by treatment as indicated. (F) Immunoblot analysis of ASC oligomerization in cross‐linked cytosolic pellets of LPS‐primed (100 ng/mL) BMDMs pretreated with MB‐3 followed by stimulation with Nigericin (10 μM). (G and H) Immunofluorescence analysis (G) and the statistics analysis (H) of ASC speckle formation in BMDMs treated as in F. White arrows: ASC speckles. Scale bar: 25 μm. ^***^ *p* < 0.001; ^****^ *p* < 0.001. One‐way ANOVA (A–C), unpaired Student's *t*‐test (D and H).

Next, LPS‐priming macrophages were treated with NLRP3 inflammasome activators such as adenosine triphosphate (ATP), Nigericin, and monosodium urate (MSU) to observe the effects of KAT2A inhibitor or knock‐down on NLRP3 inflammasome. MB‐3 treatment effectively reduced the secretion of IL‐1β upon stimulation with ATP, Nigericin, or MSU, but had no effect on TNF‐α secretion (Figures 5A–C). IL‐6 secretion was also inhibited by MB‐3 treatment in macrophages in response to LPS priming and stimulation with ATP, Nigericin, or MSU (Figures S6A–C). As expectedly, KAT2A silencing led to the decrease in production of proinflammatory cytokines such as IL‐1β and IL‐6, but not in that of TNF‐α (Figures 5D and S6D). Caspase 1 cleavage is the critical step of inflammasome activation for processing and secretion of pro‐IL‐1β.^34^ Thereby, we investigated the effects of KAT2A on the activation of NLRP3 inflammasome by detecting the protein expression of caspase 1 (p20) and IL‐1β (p17) in supernatants of cultured macrophages. It turned out that MB‐3 treatment dose‐dependently inhibited the cleavage of pro‐IL‐1β and caspase 1 protein after ATP or Nigericin stimulation (Figure 5E). ASC is the determinative factor of NLRP3 inflammasome assembly, including NLRP3‐dependent ASC oligomerization and ASC speck‐like oligomeric structure formation.^35^ Then we explored whether MB‐3 treatment affected NLRP3‐dependent ASC activation. As shown in Figure 5F, MB‐3 treatment significantly inhibited the ASC oligomerization in a dose‐dependent manner. Immunofluorescence (IF) assays demonstrated that Nigericin treatment in LPS‐primed macrophages could give rise to ASC speck‐like oligomeric structure formation, which was repressed by MB‐3 treatment (Figures 5G and H).

Next, we further observed the effect of MB‐3 on NLRP3 inflammasome activation in CIA model in vivo. The immunoblot of synovial tissues from CIA mice demonstrated that NLRP3 inflammasome activation was inhibited by MB‐3 treatment, including the decreased NLRP3 expression and cleavage of IL‐1β and caspase 1 (Figure S7). To investigate whether the therapeutic effect of MB‐3 on inflammatory tissue injury in vivo was NLRP3 inflammasome dependent, we treated mice with MCC950, a proved and effective NLRP3 inhibitor, alone or together with MB‐3 to verify the participation of NLRP3 inflammasome in macrophage responses in vivo. MB‐3 treatment had the obvious therapeutic effect on in vivo inflammatory tissue injury and IL‐1β production, which was as significant as MCC950 treatment. Additionally, cotreatment with MB‐3 and MCC950 did not showed any more superimposed effect, compared with that of MB‐3 treatment group (Figures S8A–C). These results indicate that the inhibitory effect of KAT2A in macrophage activation is largely dependent on NLRP3 inflammasome. Thus KAT2A promotes pathogenic IL‐1β secretion and tissue inflammation by promoting the priming step and subsequent inflammasome assembly.

### 2.7 KAT2A supports the metabolic reprogramming of inflammatory macrophages by suppressing NRF2 pathway

**FIGURE 6:**
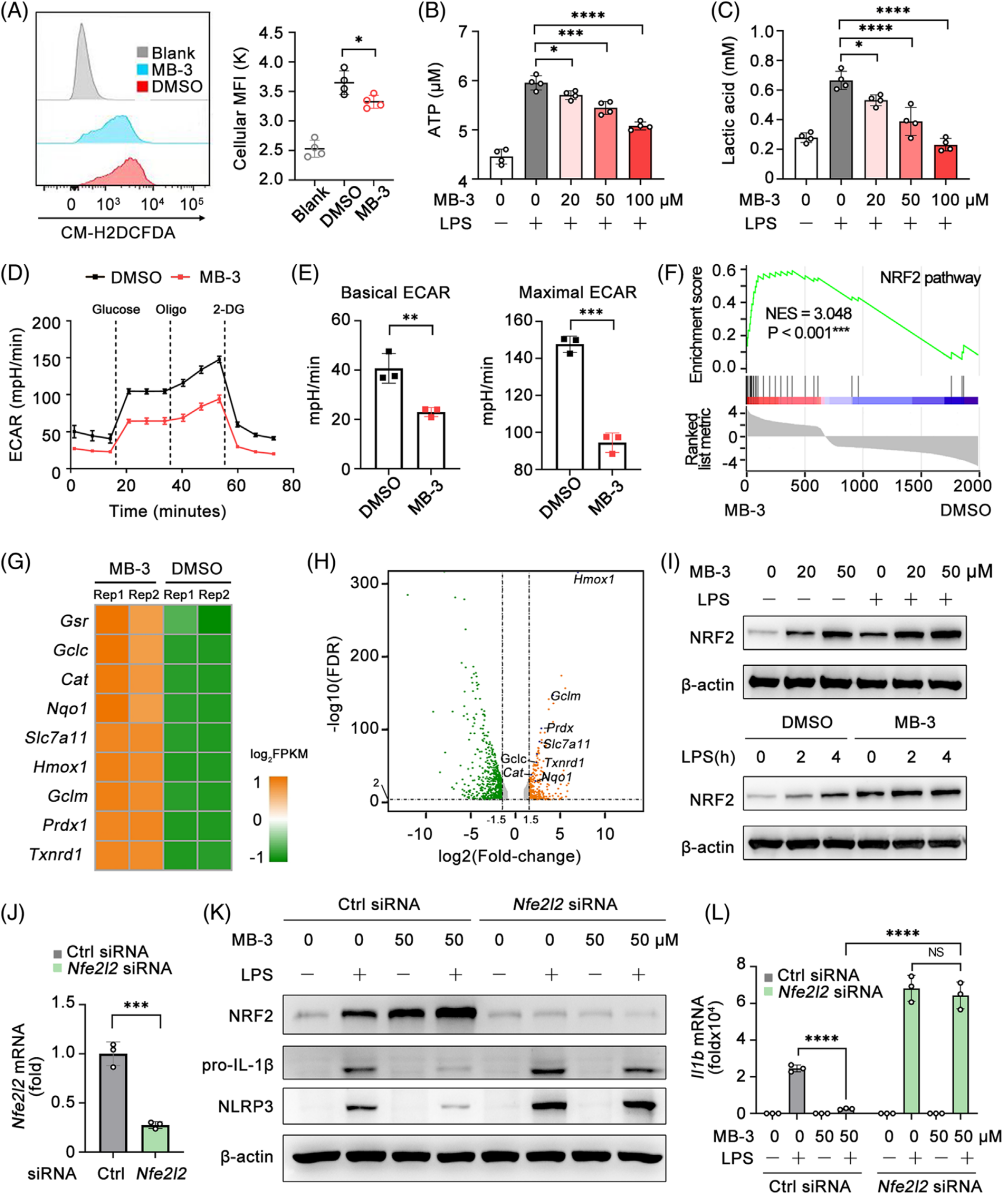
KAT2A‐mediated NRF2 pathway suppression licenses the metabolic reprogramming of inflammatory macrophages. (A) Flow cytometric analysis of cellular ROS in BMDMs pretreated with MB‐3 (50 μM) followed by LPS (100 ng/mL) stimulation for 4 h. (B and C) Intracellular ATP concentration (B) and lactic acid production (C) in BMDMs pretreated with the indicated doses of MB‐3 followed by stimulation with LPS (100 ng/mL) for 4 h. (D and E) ECAR analysis (D), basal ECAR and maximal ECAR (E) of BMDMs pretreated with MB‐3 (50 μM) followed by LPS stimulation (100 ng/mL) for 4 h. (F–H) GSEA analysis (F), heatmap of differentially expressed gene in NRF2 pathway (G) and scatter plots of gene expression in mouse BMDMs pretreated with MB‐3 (50 μM) or DMSO followed by stimulation with LPS (100 ng/mL) for 4 h (H). (I) Immunoblot analysis of NRF2 expression in BMDMs pretreated with the indicated doses of MB‐3 followed by stimulation with LPS (100 ng/mL) for 4 h. (J) Q‐PCR analysis of *Nfe2l2* (*Nrf2*) mRNA level in BMDMs transfected with control siRNA or *Nfe2l2* siRNA. (K and L) Immunoblot analysis of the indicated proteins (K) and Q‐PCR analysis of *Il1b* mRNA level (L) in BMDMs transfected as in J and then treated with the indicated doses of MB‐3 followed by LPS stimulation (100 ng/mL). ^*^ *p* < 0.05; ^**^ *p* < 0.01; ^***^ *p* < 0.001; ^****^ *p* < 0.001. One‐way ANOVA (A–C, L), unpaired Student's *t*‐test (E and J).

Reactive oxygen species (ROS) is the crucial elements for NLRP3 activation.^36^ Thereby we assessed the effect of MB‐3 on cellular ROS generation. Flow cytometry analysis showed that MB‐3 treatment significantly inhibited cellular ROS generation in BMDMs stimulated with LPS (Figure 6A). Macrophages undergo metabolic reprogramming to facilitate macrophage effector function in response to various inflammatory stimuli, especially during NLRP3 inflammasome activation. In LPS‐activated macrophages, distinct breaks in the tricarboxylic acid cycle lead to impaired mitochondria respiration and glycolysis enhancement.^37^ Disruption of glycolytic flux impairs NLRP3‐dependent IL‐1β production.^38^ In this way, we evaluated the glycolysis level by detecting two main glycolysis indicators, ATP generation, and lactic acid concentration. As shown in Figures 6B and C, LPS stimulation led to the enhanced production of ATP and lactic acid, which was decreased by MB‐3 treatment, indicating that LPS‐triggered switch from mitochondria respiration to anaerobic glycolysis was impaired by MB‐3. To confirm the effect of MB‐3 on glycolysis, we assessed the cellular glycolysis level by directly determining the extracellular acidification rate (ECAR) in LPS‐stimulated macrophages with or without MB‐3 treatment (Figure 6D). The results turned out that MB‐3 treatment remarkably led to the decrease in both basal and maximal ECAR in macrophages, confirming the impaired glycolysis level after MB‐3 treatment (Figures 6D and E).

In order to uncover the underlying mechanisms of KAT2A on macrophage metabolism reprogramming, we reanalyzed the data of RNA‐seq from LPS‐activated BMDMs pretreated with MB‐3 or DMSO, and found that differentially expressed gene were enriched in NRF2 pathway (Figure 6F), the main antioxidant response controlling cellular metabolic phenotype and stress‐induced ROS.^36^ Furthermore, NRF2 has reported to regulate NLRP3 inflammasome in the metabolic and transcriptional manners.^39^, ^40^ Further analysis of RNA‐seq data found that many upregulated genes in MB‐3‐treated BMDMs were related to NRF2 function and transcription activity, consistent with the GSEA result (Figures 6F–H). We detected the expression of NRF2, encoded by *Nfe2l2* gene in BMDMs treated with or without MB‐3. MB‐3 treatment induced the upregulation of NRF2 protein expression in LPS‐activated BMDMs (Figures 6I and S9). Next, we studied the mechanisms underlying the upregulation of NRF2 protein expression mediated by MB‐3. Previous work has showed that NRF2 protein expression was tightly regulated by glucose metabolite itaconate‐mediated alkylation of KEAP1 (kelch‐like ECH associated protein 1).^40^ Thus, we speculated whether KAT2A influenced the protein stability of NRF2 dependent on itaconate/KEAP1 pathway. We isolated BMDMs from *Irg1^fl/fl^* control mice and macrophage‐conditional IRG1 knockout (*Irg1^fl/fl^Lyz2‐cre^+^*) mice (Figure S10A). IRG1 knockout mice lacked the ability to produce itaconate and impaired immunosuppressive function.^40^, ^41^ While KAT2A inhibition resulted in the upregulation of NRF2 in control BMDMs, it had no effects on NRF2 expression in IRG1‐deficient BMDMs unless the re‐supplementation of 4‐OI, a metabolite derivative of itaconate (Figure S10B). These results suggested the regulatory effect of MB‐3 on NRF2 protein level might be largely dependent on metabolism rewiring of itaconate, which was recently identified as the major physiological regulator of KEAP1/NRF2 activity and effector function of inflammatory macrophages. Next, NRF2 expression was silenced with the specific siRNA in BMDMs (Figure 6J), and then NRF2‐slienced BMDMs and control BMDMs were treated with MB‐3 to investigate the participation of NRF2 on transcription and protein synthesis of IL‐1β. Although MB‐3 had obvious inhibitory effect in control BMDMs, it had no effect on the NLRP3 inflammasome priming including NLRP3 as well as pro‐IL‐1β protein synthesis in NRF2‐silenced BMDMs (Figures 6K and L). These results indicate that the inhibitory effect of MB‐3 on inflammatory responses relies on NRF2 upregulation and its anti‐inflammatory effects. Last, we detected the ECAR level in BMDMs with *Kat2a* silencing and found the similar reduction of basal ECAR and maximal ECAR (Figure S11). Our data show KAT2A is required for the macrophage glycolysis reprogramming for NLRP3 activation through inhibiting NRF2 expression and activity.

### 2.8 KAT2A coordinates histone acetylation with NRF2 transcription activity for *Il1b* and *Nlrp3* transcription

**FIGURE 7:**
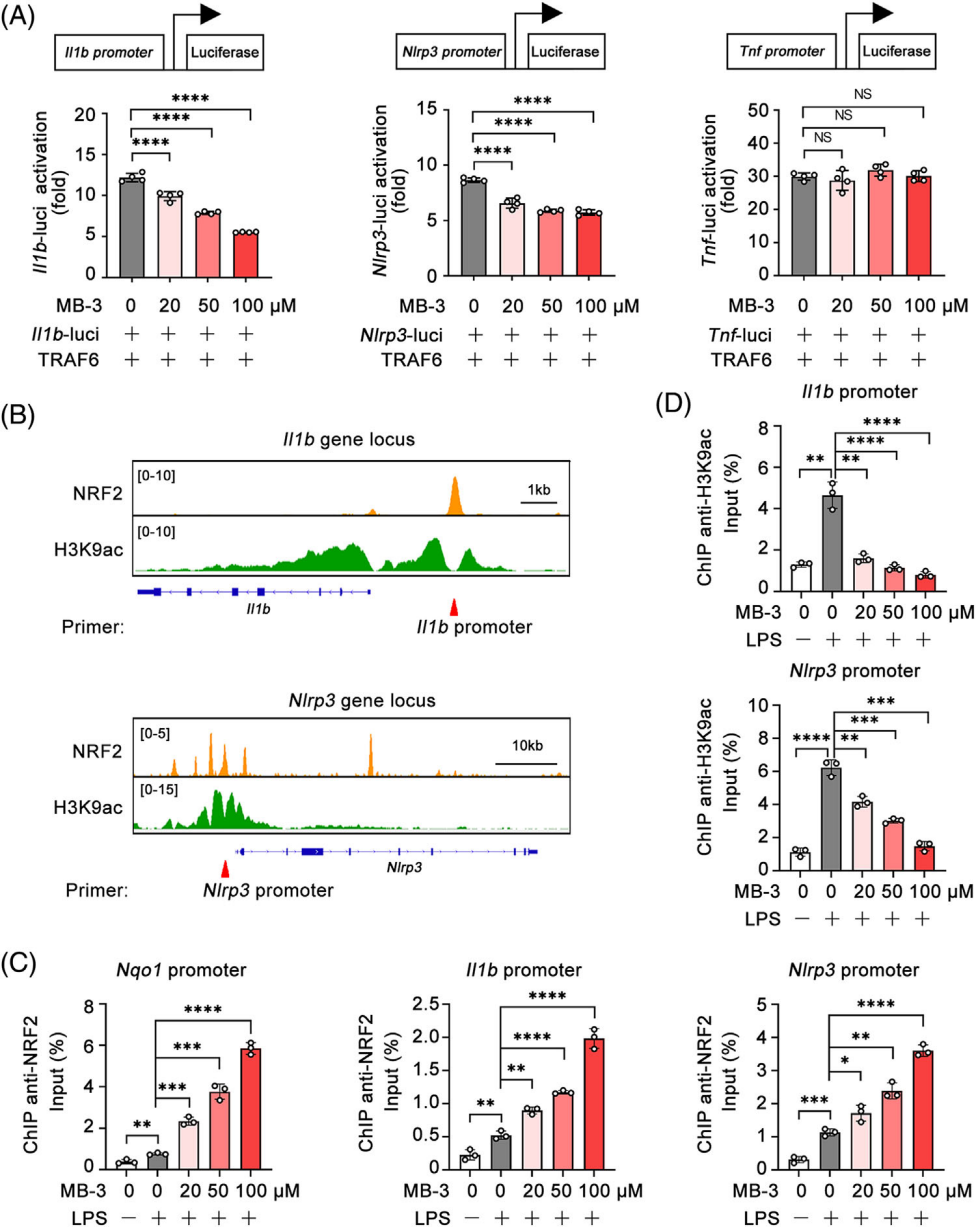
KAT2A coordinates histone acetylation with NRF2 transcription activity to promote *Il1b* and *Nlrp3* expression. (A) Dual‐luciferase reporter analysis of the indicated promoter activation triggered by TRAF6 in HEK293T cells treated with the indicated amounts of MB‐3. (B) IGV analysis of NRF2 (GSE72946) and H3K9ac (GSE113226) signal in the gene locus of *Il1b* and *Nlrp3*. (C and D) ChIP analysis of NRF2 (C) and H3K9ac enrichment (D) at the indicated gene promoter in BMDMs pretreated with MB‐3 followed by stimulation with LPS (100 ng/mL) for 4 h. ^*^ *p* < 0.05; ^**^ *p* < 0.01; ^***^ *p* < 0.001; ^****^ *p* < 0.001. One‐way ANOVA (A, C, and D).

**FIGURE 8:**
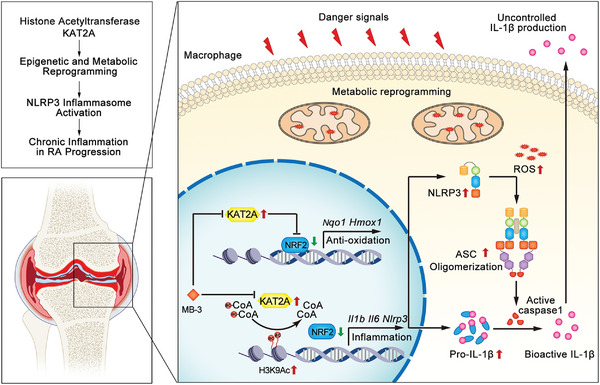
Graphical representation of KAT2A in licensing the epigenetic and metabolic reprogramming of inflammatory macrophages. In synovial tissues of RA patients, the increased expression of KAT2A promotes the inducible transcription of *Il1b* and *Nlrp3* genes by catalyzing H3K9ac and limiting NRF2 activity, thereby leading to uncontrolled IL‐1β production and inflammatory injury in RA progression.

To reveal the mechanism underlying the suppression of *Il1b* and *Nlrp3* transcription mediated by KAT2A inhibition, we performed the double luciferase reporter gene assays with transfection of luciferase reporter plasmids contained *Il1b* promoter, *Nlrp3* promoter or *Tnf* promoter, respectively. MB‐3 treatment dose‐dependently inhibited the TRAF6‐induced *Il1b* and *Nlrp3* promoter activation, but had little influence on *Tnf* promoter activation (Figure 7A), suggesting that MB‐3 controlled *Il1b* and *Nlrp3* gene expression at the transcription level. Then, we investigated whether this effect was mediated by NRF2, which had been proven as the master regulator of *Il1b* transcription.^40^, ^42^, ^43^ Chromatin immunoprecipitation‐sequencing (ChIP‐seq) assays from public database showed that NRF2 specifically bound to the promoters of *Il1b* and *Nlrp3* in LPS‐stimulated macrophages, where NRF2 influenced the H3K9ac level^44^, ^45^ (Figure 7B). We guessed whether MB‐3 regulated gene expression through two separate approaches, inhibiting KAT2A‐mediated histone H3K9ac as well as promoting NRF2‐mediated transcription repression. In order to confirm our suspect, we performed ChIP‐qPCR assays using NRF2 antibody or H3K9ac antibody. As shown in Figure 7C, MB‐3 treatment markedly promoted the NRF2 enrichment on the *Nqo1* (NADPH quinone dehydrogenase 1) promoter, as the positive control. And MB‐3 treatment also enhanced the enrichment of NRF2 to both the promoters of *Il1b* and *Nlrp3*, indicating the elevation in NRF2 transcription repressor activity (Figure 7C). Meanwhile, the results of ChIP‐qPCR using H3K9ac antibody showed that KAT2A inhibition by MB‐3 also decreased the H3K9ac level on the promoters of *Il1b* and *Nlrp3*, manifesting the loss of transcription activation capability mediated by MB‐3 treatment (Figure 7D). To sum up, our study demonstrate that MB‐3 coordinates the repressed histone H3K9ac modification with the enhanced NRF2 transcription repressor activity for controlling *Il1b* and *Nlrp3* mRNA expression. To sum up, KAT2A drives the abnormal activation of NLRP3 inflammasome and excessive IL‐1β production in inflammatory macrophages by suppressing NRF2‐mediated metabolic reprogramming, resulting in the progressive articular damage in RA (Figure 8).

## 3 DISCUSSION

Our work reveals the pathogenic role of KAT2A in the progress of RA, which is ameliorated by its selective inhibitors. The pro‐IL‐1β and NLRP3 protein expression level is considered as a rate‐limiting element for NLRP3 inflammasome assembly and activation.^46^ Using CIA model and LPS‐triggered systemic inflammation model, we demonstrate that KAT2A is essential for NLRP3 inflammasome activation by promoting the transcription of *Il1b* and *Nlrp3*. Additionally, Kim et al.^47^ reported that in IL‐1β‐treated HepG2 cells, KAT2A bound to the promoter of IL‐1β‐responsive genes for mediating the transcription activation via H3K9ac, indicating KAT2A might be also indispensable for IL‐1β downstream and effectors. A recent work found that in systemic lupus erythematosus (SLE), KAT2A was abnormally upregulated, and modulated the expression and acetylation of cyclic GMP‐AMP synthase.^48^ Clinical data identified that NLRP3 was hyperactivated in macrophages of SLE patients and its dysregulation was positively correlated with SLE disease activity index.^49^ In this way, the enhanced inflammasome activation by KAT2A might also contribute to the pathogenesis of SLE. Collectively, we propose that targeting KAT2A exhibits the therapeutic potential in several inflammatory diseases including RA, SLE, and so on.

Previous studies demonstrated that KAT2A controlled glucose and lipid metabolic pathways and its dysfunction resulted in metabolic diseases. In primary skeletal muscle cells, KAT2A controls nutrient and energy homeostasis through directly interacting with peroxisome proliferator‐activated receptor γ coactivator 1 (PGC‐1) and mediating its lysine acetylation. KAT2A silencing ablates PGC‐1 acetylation but helps its transcriptional function for mediating the expression of several metabolic genes, including Glucose Transporter Type 4, indicating a role of KAT2A in inhibiting insulin‐mediated glucose transport.^50^ Besides, together with its homolog PCAF, KAT2A is involved in brown adipogenesis through epigenetically promoting PR/SET Domain 16 expression and thus facilitating peroxisome proliferator activated receptor γ transcript elongation.^51^ However, no evidence had been found that KAT2A could influence the cellular glycolysis level in macrophages. Our work found that KAT2A inhibition or knockdown inhibited the metabolic switch from OXPHOS to glycolysis during inflammation, thereby impaired macrophage effector function. We showed that the immune‐stimulatory function of KAT2A was NRF2 pathway dependent. Nevertheless, the mechanism of KAT2A in regulation of NRF2 activity was largely unknown. Increasing evidence demonstrates that NRF2 regulates *Il1b* transcription and NLRP3 inflammasome activation in direct or indirect manners.^39^, ^43^, ^52^ As a master regulator of cellular antioxidative response, the expression and activity of NRF2 is tightly regulated by several mechanisms, for example, the interaction of autophagy substrate p62 and KEAP1. An overproduction of p62 disrupted the physical interaction of NRF2 with KEAP1, resulting in NRF2 protein stabilization and enhanced transcriptional induction of downstream antioxidative and anti‐inflammatory genes.^53^ Recently, NRF2/KEAP1 axis was reported to control inflammatory responses through a negative feedback loop involving metabolite itaconate and IFN.^40^ In our study, KAT2A inhibition significantly promoted the protein expression of NRF2, which inferred that KAT2A regulated NRF2 expression at the posttranslational level. Additionally, we found that IRG1‐mediated itaconate production was essential for the upregulation of NRF2 by MB‐3. Therefore, we preliminarily conclude that KAT2A inhibitor‐mediated cellular metabolism reprogramming has the feedback regulatory function on NRF2 expression, and thus amplifies its antioxidant and anti‐inflammatory effects.

Based on the experimental data discussed above, we propose a model how KAT2A promotes the NLRP3‐dependent IL‐1β production and subsequent inflammatory responses. KAT2A promotes the *Il1b* and *Nlrp3* transcription through two approaches including histone H3K9ac modification and limiting NRF2 transcription repressor activity. As the rate‐limiting element of NLRP3 inflammasome activation, NLRP3 is indispensable for ASC oligomerization and caspase 1 cleavage, thus promoting the processing pro‐IL‐1β into bioactive IL‐1β and secretion. Besides immune system diseases, uncontrolled inflammasome pathway was found to participate in the pathogenesis of other common disorders, such as cardiovascular diseases, tumor progress, and neurodegenerative diseases.^54^ These finding means targeting KAT2A may be also effective in the treatment of other diseases caused by NLRP3 inflammasome.

However, this study does not elucidate the detailed molecular mechanism how KAT2A influences macrophage metabolism and itaconate production. Although we performed loss‐of‐function assays using IRG1 knockout cells to access the role of KAT2A and MB‐3, the actual cellular itaconate concentration was hard to measure without available commercial assay kit. In addition, we focused the proinflammatory effects of KAT2A in macrophage‐mediated arthritis. Nevertheless, it could not exclude whether KAT2A had the regulatory role in other pathogenic immune cells for arthritis. It is tempting to evaluate the biological importance of KAT2A with different immune cell conditional knockout mice (e.g., Lyz2‐Cre, CD4‐Cre, or CD19‐Cre). Finally, this work identified two proinflammatory genes (*Il1b* and *Nlrp3*) as the direct target genes of KAT2A in macrophages. However, the impact of KAT2A in gene transcription was extensive, according to RNA‐seq results. With ChIP‐seq or other genome‐wide epigenetic sequencing, the directed regulatory networks about the relationships between KAT2A and other target genes in immune system would be fully clarified.

Finally, our work provides a novel insight of epigenetic acetyltransferase KAT2A into the regulation of NLRP3 activation and IL‐1β production, which exhibits potential therapeutic approach for RA and relevant inflammatory diseases.

## 4 MATERIALS AND METHODS

### 4.1 Human participant research

Thirty‐eight subjects (28 RA cases and 10 health controls) from Changhai Hospital (Shanghai, China) were enrolled as described before.^55^ Briefly, RA patients fulfilled the 2010 criteria of the American College of Rheumatology and the European Union League Against Rheumatism, and classified as ARA or SRA by 28‐joint disease activity score (DAS28) (ARA score: ≥2.6, SRA < 2.6). Health controls were not suffering from severe cardiovascular diseases, liver and kidney dysfunction, malignant tumor, and other autoimmune or musculoskeletal disorders. PBMCs and synovial tissues were immediately isolated from RA cases or controls. This study was approved by the Ethics Committee of Changhai Hospital (Approval No. CHEC2020105).

### 4.2 Reagents and antibodies

MB‐3 (M2449), complete Freund's adjuvant (CFA) (F5881), and LPS (E.coli 0111:B4) were purchased from Sigma–Aldrich. Chicken Type II Collagen (20088) was from Chondrex. ChIP‐grade protein G magnetic beads (9006) and cell lysis buffer (9803) were from Cell Signaling Technology (CST). Nigericin (tlrl‐nig), ATP (tlrl‐atpl), and MSU (tlrl‐msu) were from InvivoGen. Mouse IL‐1β, TNF‐α, and IL‐6 ELISA kits were from R&D Systems. Antibodies against KAT2A (3305), IL‐1β (12242), NRF2 (12721), IRG1 (19857), Acetyl‐Histone H3 Lys9 (9649), and β‐Actin (3700) were from CST. Antibodies against NLRP3 (AG‐20B‐0014B), caspase 1 (AG‐20B‐0042B), and ASC (AG‐25B‐0006PF) were from AdipoGen Life Sciences. Antibodies against CD45‐BV605 (2D1), CD4‐BV421 (GK1.5), CD8a‐BV510 (53‐6.7), CD11b‐BV510 (M1/70), F4/80‐FITC (BM8), CD44‐APC (IM7), CD62L‐PE (MEL‐14), PD‐1‐APC (29F.1A12), CXCR5‐PE (J252D4), IFN‐γ‐APC (XMG1.2), and IL‐17a‐PE (TC11‐18H10.1) were from Biolegend. Antibody against FoxP3‐PE (FJK‐16s) was obtained from eBioscience.

### 4.3 Mice

C57BL/6 mice (6−10 weeks old) and DBA/1 mice (10−12 weeks old) were purchased from Joint Ventures Sipper BK Experimental Animal Company (Shanghai, China) and Gempharmatech Company (Jiangsu, China), respectively. Mice were bred in specific pathogen‐free conditions. All mice experiments were randomized and both male mice and female mice were used. Animal experiments were approved by the Scientific Investigation Committee of Tongji University School of Medicine (Ethics NO. TJTJ00320101) and carried out in accordance with the Guide for the Care and Use of Laboratory Animals.

### 4.4 CIA model

DBA/1 mice were used to establish CIA model as described before.^56^ On day 0, chicken type II collagen emulsified in CFA (1:1, v/v) was injected into mouse tail base for the first immunization. On day 21, a booster immunization was performed as before. Symptoms of arthritis presented around on day 28. All CIA mice were treated with MB‐3 (10 mg/kg body weight, i.p.) or DMSO on alternate days. And the severity of redness and swelling in the wrist and paw in a scale of 0−4 was evaluated from day 21 to day 49. Knee joints and paws were fixed in paraformaldehyde for further detection of micro‐CT, H&E, and Safranin O staining. Sera were freshly collected for ELISA assays. Spleen and PLNs were collected and prepared as single‐cell suspension for analyzing immune cell subtypes via FACS assays.

### 4.5 LPS‐induced endotoxin shock and peritonitis model

For endotoxin shock model,^27^ C57BL/6 mice were pretreated with MB‐3 (10 mg/kg body weight, i.p.) or DMSO and then injected i.p. with LPS (12 mg/kg body weight). Sera were collected 8 h after injection for ELISA assays. Lung tissues were fixed in paraformaldehyde for H&E staining. For peritonitis model,^57^ C57BL/6 mice were pretreated with MB‐3 (10 mg/kg body weight, i.p.) or DMSO and then injected i.p. with LPS (100 ng/mouse). 8 h later, the immune cells from peritoneal lavage fluids were collected for quantitative PCR (Q‐PCR) assays or FACS assays.

### 4.6 Flow cytometry and intracellular cytokine staining

Single‐cell suspensions were prepared from spleens, pLNs, or peritoneal lavage fluids for FACS assays via LSRFortessa (BD Biosciences) and further for statistical analysis via FlowJo software. Cells were stained with fluorochrome‐labeled antibodies following incubation with anti‐CD16/CD32 to block Fc receptors. For intracellular cytokine (IFN‐γ and IL‐17a) detection, cells were incubated with anti‐CD16/32 and surface staining 6 h after Cell Activation Cocktail (with Brefeldin A) (Biolegend) stimulation. Then, cells were subjected to fixation/permeabilization kit (BD Biosciences) for intracellular cytokines staining. FoxP3/Transcription Factor Staining Buffer Set (Thermo) was used for detection of FoxP3 expression according to the instruction.

### 4.7 Cell culture and stimulation

BMDMs were prepared and cultured as described previously.^58^ Briefly, bone marrow cells were extracted from the leg bones and differentiated in RPMI‐1640 (Gibco) with 10% (v/v) FBS (Gibco) containing M‐CSF (20 ng/mL) (PeproTech). The medium was substituted with fresh medium after 3 days. On day 6 or 7, BMDMs were harvested and replated for experiments. For macrophage stimulation, LPS was used at 100 ng/mL for the indicated time unless otherwise specified. For NLRP3 inflammasome activation, macrophages were primed with LPS (100 ng/mL) for 3 h and then stimulated with nigericin (10 μM) and ATP (5 mM) for 1 h or MSU (200 μg/mL) for 6 h. HEK293T cell line was purchased from American Type Culture Collection and cultured as described previously.^58^

### 4.8 RNA isolation and Q‐PCR assay

Total RNA was isolated and purified via TRIzol reagent (Thermo) according to the direction. Q‐PCR assays were performed using SYBR Green reagent (Takara) via LightCycler 480 (Roche). Data were normalized to mouse *Actb* or human *ACTB* expression. The primers for mouse genes or human genes were listed in Tables S1 and S2, respectively.

### 4.9 RNA‑seq

RNA‐seq was performed as described before.^55^ Briefly, total RNA was obtained using RNeasy mini kit (Qiagen). Strand‐specific libraries for RNA‐seq were prepared using the TruSeq stranded total RNA sample preparation kit (Illumina), quantified by Qubit 2.0 Fluorometer (Life Technologies) and validated for insert size by 2100 bioanalyzer (Agilent). Cluster was generated by cBot with the library diluted to 10 pM and subjected to sequencing using NovaSeq 6000 (Illumina). The library construction and sequencing were performed by Shanghai Biotechnology Corporation. RNA‐seq data are available in the GEO database (No: GSE205238).

### 4.10 RNA interference

The siRNAs targeting *Kat2a* (L‐040665‐01‐0010) and nontargeting control (D‐001810‐10‐05) were from Dharmacon. The siRNAs targeting *Nrf2* (sc‐37049) and control siRNA (sc‐37007) were from Santa Cruz Biotechnology. Macrophages were transfected with the indicated siRNAs by Lipofectamine RNAiMAX (Invitrogen), according to the manufacturer's directions.

### 4.11 Dual‐luciferase reporter gene assay

HEK293T cells were transfected with combinations of plasmids using JetPEI (PolyPlus) under manufacturer's instructions. After 24 h, luciferase activity was detected by a dual‐luciferase reporter assay (Promega). Data were normalized for transfection efficiency by the division of firefly luciferase activity with that of *Renilla* luciferase.

### 4.12 Immunoblot

Total proteins from tissues or cells were extracted by cell lysis buffer with additional protease inhibitor cocktail (Calbiochem) and PMSF (Millipore). Protein concentration was measured by the BCA reagent kit (Thermo). Proteins were subjected to immunoblot assay as previously reported.^27^

### 4.13 ASC oligomerization and ASC speck formation assays

For immunoblot of ASC oligomerization, BMDMs were lysed with Triton Buffer (pH 7.5 50 mM Tris HCl, 150 mM NaCl, 0.5% Triton X‐100, and 0.1 mM PMSF) for 10 min on ice. The cell lysates were centrifuged at 6,000×*g* for 15 min at 4°C and then resuspended in Triton Buffer and disuccinimidyl suberate (2 mM) (Sangon Biotech). After incubation for 30 min at 37°C for cross‐linking, cell pellets were washed by Triton Buffer for two times. Then cell lysates were centrifuged at 12,000×*g* for 15 min at 4°C, and were redissolved in 1× SDS loading buffer (Sangon Biotech) for immunoblot assays. For IF analysis of ASC speck formation, BMDMs washed with cold PBS three times, fixed in paraformaldehyde (4%, v/v) for 10 min, permeabilized with Triton X‐100 (0.1%, v/v) for 15 min, and blocked with BSA (3%, v/v). Cells were then stained with ASC antibody (1:200) overnight at 4°C and stained with Alexa fluor 488‐labeled secondary antibody (1:200) (Abcam) for 1 h at room temperature. Last, DAPI was used to stain cell nuclei. Cells were visualized using TCS SP2 confocal laser microscope (Leica).

### 4.14 Seahorse metabolic analysis

The ECAR was measured with Seahorse XFe96 Extracellular Flux Analyzer (Agilent), according to the manufacturer's instructions. Briefly, macrophages were obtained as before, then plated in Seahorse 96‐well plates for LPS stimulation. Glycolytic stress test was performed using the following injection strategy, 25 mM glucose, 1 mM oligomycin (OM), and 100 mM 2‐DG from Seahorse XF Glycolytic Rate Assay Kit (Seahorse, Agilent). Basal and maximal ECAR were calculated as follows: basal ECAR = ECAR_before OM_ − ECAR_after 2‐DG_; and maximum glycolytic capacity = ECAR_after OM_ − ECAR_after 2‐DG_.

### 4.15 Metabolite assay

The lactate production was determined with Lactate Colorimetric Assay Kit II (Biovision) under manufacturer's direction. The intracellular ATP concentrations was determined by ATP Determination Kit (Beyotime Biotech).

### 4.16 ChIP assay

ChIP assays were performed using Chromatin Immunoprecipitation Assay Kit (Millipore) and quantified using Q‐PCR. Data were normalized by input DNA for % input each sample. The primers for ChIP assays were shown in Table S3.

### 4.17 Statistical analysis

Statistical analyses were performed by GraphPad Prism software 9.0. The data were taken from at least three independent experiments (mean ± SD). For comparison of two groups, unpaired Student's *t*‐test was performed. For comparison of more than two groups, one‐ or two‐way ANOVA was performed. Difference was considered to be significant when *p* < 0.05.

## AUTHOR CONTRIBUTIONS

Yunkai Zhang, Ying Gao, Yingying Ding, Yuyu Jiang, and Huiying Chen performed experiments. Yunkai Zhang and Ying Gao designed experiments, analyzed data, performed bioinformatics analysis, and drafted the manuscript. Xingguang Liu and Zhenzhen Zhan designed the research, analyzed data, and revised the manuscript. All authors approved the final manuscript.

## CONFLICT OF INTEREST STATEMENT

The authors declare no competing interests.

## ETHICS STATEMENT

The clinical experiment in this study was approved by the Ethics Committee of Changhai Hospital, Naval Medical University (Approval No. CHEC2020105). Written informed consent has been obtained from all patients. Animal experiments were approved by the Scientific Investigation Committee of Tongji University School of Medicine (Ethics No. TJTJ00320101) and carried out in accordance with the Guide for the Care and Use of Laboratory Animals.

## Supporting information

Supporting Information

## Data Availability

Data supporting the present study are available from the corresponding author upon reasonable request.
