# Supplementary material for: Targeting KAT2A inhibits inflammatory macrophage activation and rheumatoid arthritis through epigenetic and metabolic reprogramming
Source: MedComm (2020). 2023 Jun 11;4(3):e306. doi: 10.1002/mco2.306 (PMC10258526; doi:10.1002/mco2.306)
Supplement: Supplementary file 1 — Supporting Information [file MCO2-4-e306-s001.docx]

**Supplementary information for**

**Targeting KAT2A inhibits inflammatory macrophage activation and rheumatoid arthritis through epigenetic and metabolic reprogramming**

Yunkai Zhang^1,2,#^, Ying Gao^3,4,#^, Yingying Ding^1,#^, Yuyu Jiang^1^, Huiying Chen^1^, Zhenzhen Zhan^4,5,*^, Xingguang Liu^1,2,*^

^1^Department of Pathogen Biology, Naval Medical University, Shanghai 200433, China;

^2^National Key Laboratory of Medical Immunology, Naval Medical University, Shanghai 200433, China;

^3^Department of Rheumatology, Changhai Hospital, Naval Medical University, Shanghai 200433, China;

^4^Key Laboratory of Arrhythmias of the Ministry of Education of China, Research Center for Translational Medicine, Shanghai East Hospital, Tongji University School of Medicine, Shanghai 200120, China;

^5^Department of Liver Surgery, Shanghai Institute of Transplantation, Renji Hospital, Shanghai Jiao Tong University School of Medicine, Shanghai 200127, China.

^#^Yunkai Zhang, Ying Gao, and Yingying Ding contributed equally to this work.

***Correspondence**

Xingguang Liu, Department of Pathogen Biology, Naval Medical University, Shanghai 200433, China. E-mail: liuxg@immunol.org

Zhenzhen Zhan, Key Laboratory of Arrhythmias of the Ministry of Education of China, Research Center for Translational Medicine, Shanghai East Hospital, Tongji University School of Medicine, Shanghai 200120, China. E-mail: zhanzz@tongji.edu.cn

**Short title**: KAT2A inhibitor alleviates rheumatoid arthritis

**This file includes:** Figure S1-11, Table S1-3.


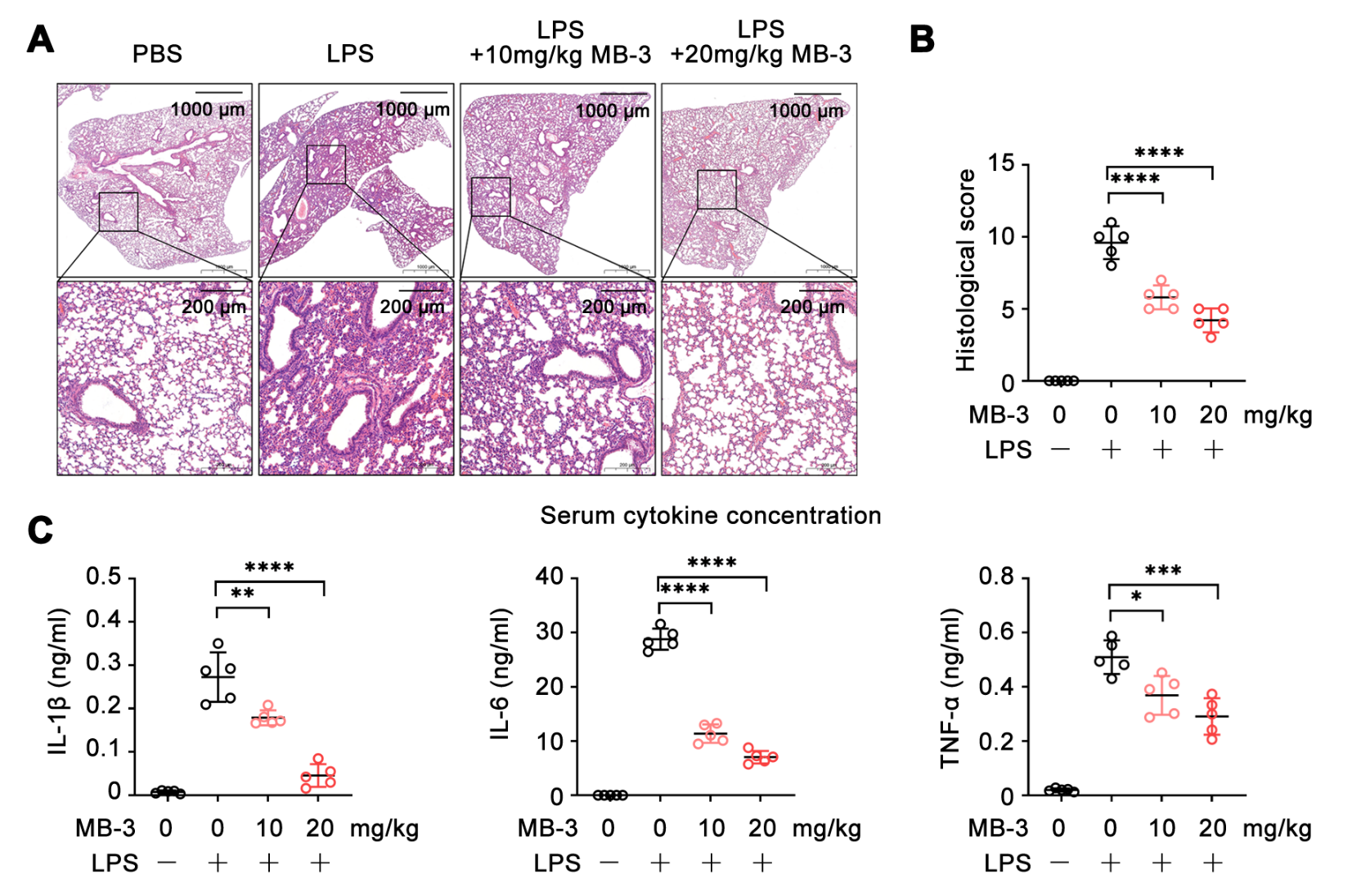


**Figure S1. KAT2A inhibitor suppresses LPS-induced injury and inflammation in lung *in vivo*.**

(**A-B**) H&E staining (**A**) and histological scores (**B**) of lung tissues from the wild type mice pre-treated with the indicated amount of MB-3 followed by the intraperitoneal injection with LPS (12 mg/kg body weight) (n = 5 mice per group). Scale bar: 1000 μm (upper) or 200 μm (lower). (**C**) ELISA analysis of concentration of the indicated cytokines in the sera as in **A** (n = 5 mice per group). ^*^*P* < 0.05; ^**^*P* < 0.01; ^***^*P* < 0.001; ^****^*P* < 0.001. One-way ANOVA (**B-C**).


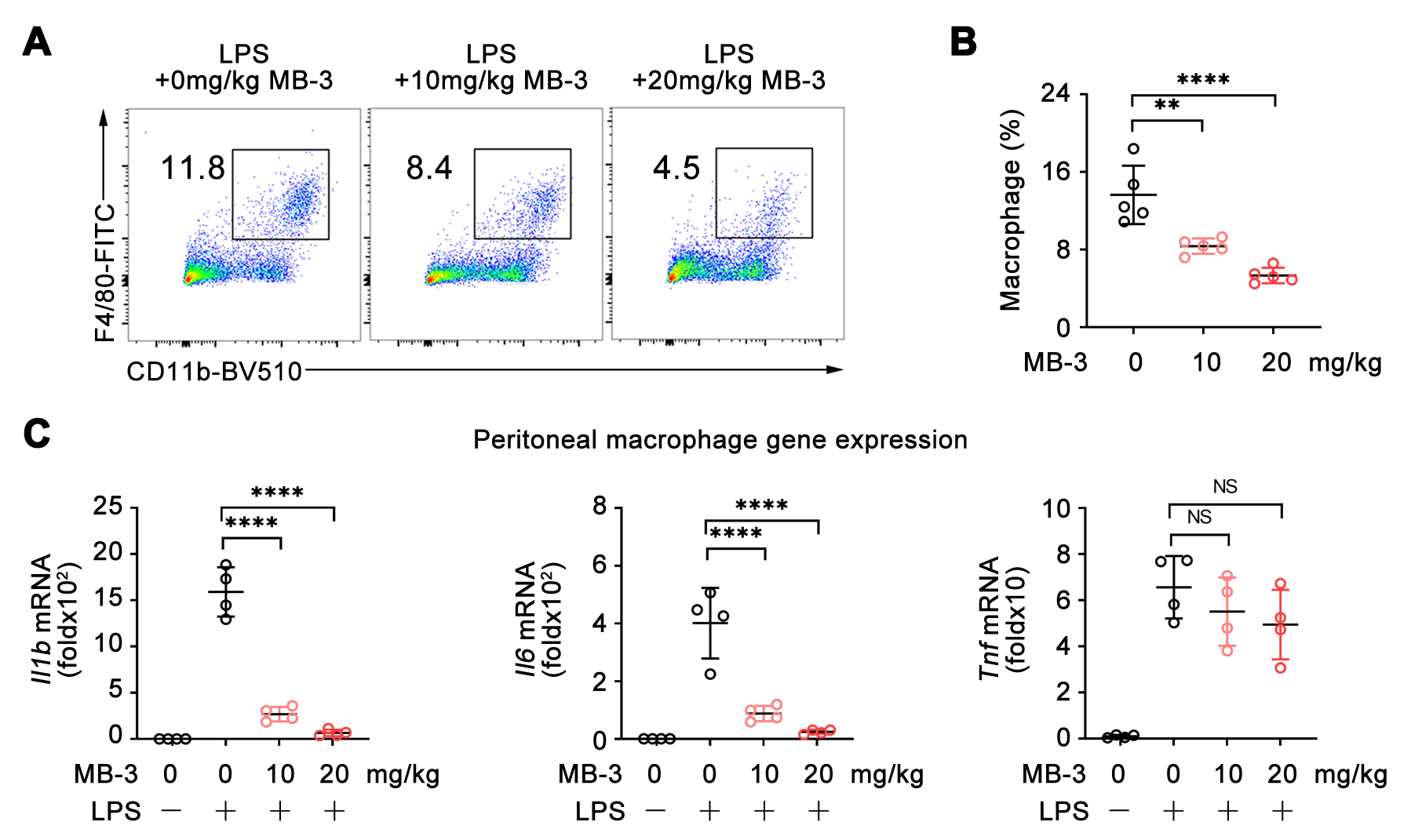


**Figure S2. KAT2A inhibitor suppresses LPS-induced peritonitis *in vivo*.**

(**A-B**) Flow cytometric analysis (**A**) and the statistics analysis (**B**) of the percentages of macrophages in peritoneal lavage fluids from wild type mice pre-treated with the indicated amounts of MB-3 followed by the intraperitoneal injection with LPS (100 ng/mouse) (n = 5 mice per group). (**C**) Q-PCR analysis of the indicated cytokine mRNA levels in peritoneal macrophages as in **A** (n = 4 mice per group). ^**^*P* < 0.01; ^****^*P* < 0.001. One-way ANOVA (**B-C**).


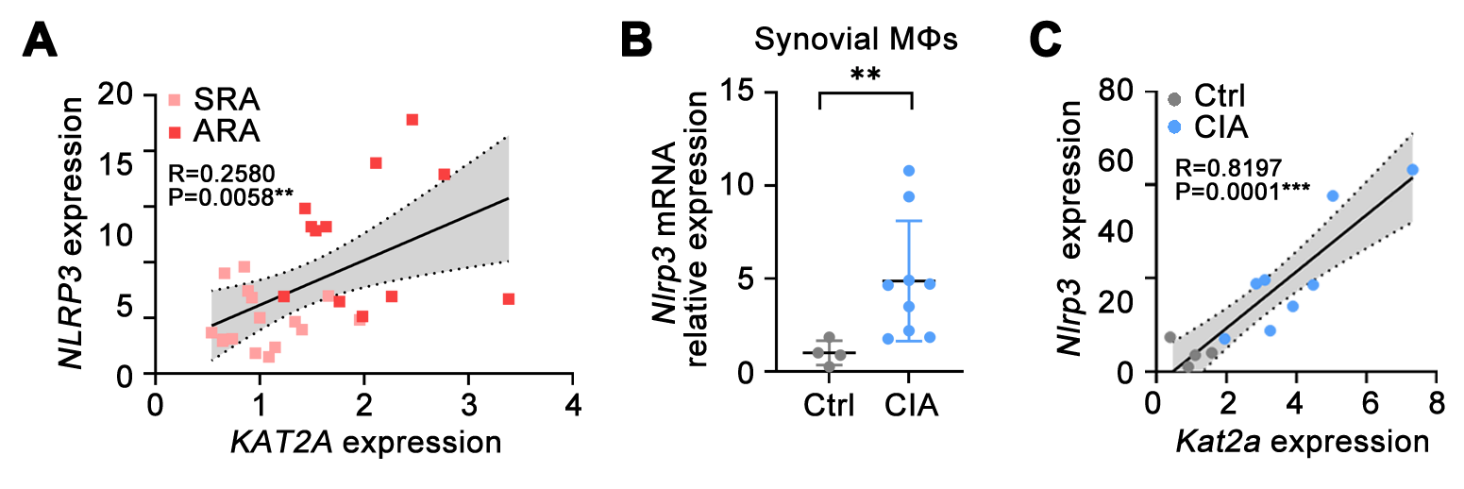


**Figure S3. The correlation between KAT2A and NLRP3 in human and mouse during arthritis.**

(**A**) Correlation analysis of *KAT2A* mRNA level with *NLRP3* mRNA level in PBMCs from SRA and ARA patients (n = 28). (**B**) Q-PCR analysis of *Nlrp3* mRNA level in synovial macrophages from CIA model mice at day 28 after the first immunization (n = 4-9 mice per group). (**C**) Correlation analysis of *Kat2a* mRNA level with *Nlrp3* mRNA level in synovial macrophages from CIA model mice as in **B**. ^**^*P* < 0.01. Unpaired Student's t test (**B**), simple linear regression (**A**, **C**).


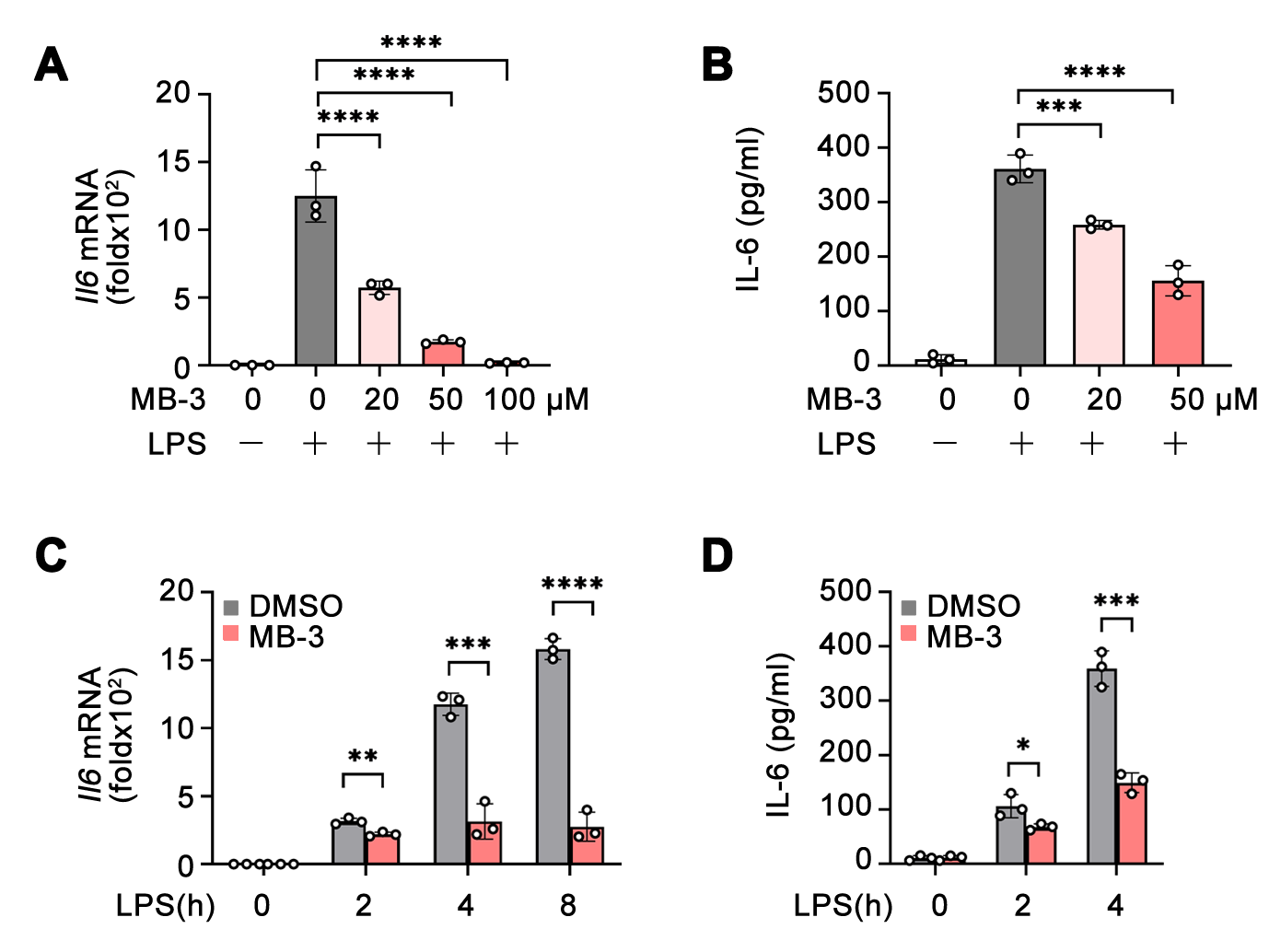


**Figure S4. KAT2A promotes LPS-triggered IL-6 transcription and secretion.**

(**A-B**) Q-PCR analysis of *Il6* mRNA (**A**) and ELISA analysis of IL-6 concentration in supernatants (**B**) of BMDMs pre-treated with the indicated amounts of MB-3 followed by stimulation with LPS for 4 h. (**C-D**) Q-PCR analysis of *Il6* mRNA (**C**) and ELISA analysis of IL-6 concentration in supernatants (**D**) of BMDMs pre-treated with MB-3 (50 μM) followed by stimulation with LPS for the indicated times. ^*^*P* < 0.05; ^**^*P* < 0.01; ^***^*P* < 0.001; ^****^*P* < 0.001. One-way ANOVA (**A**, **B**), unpaired Student's t test **(C**, **D)**.

**
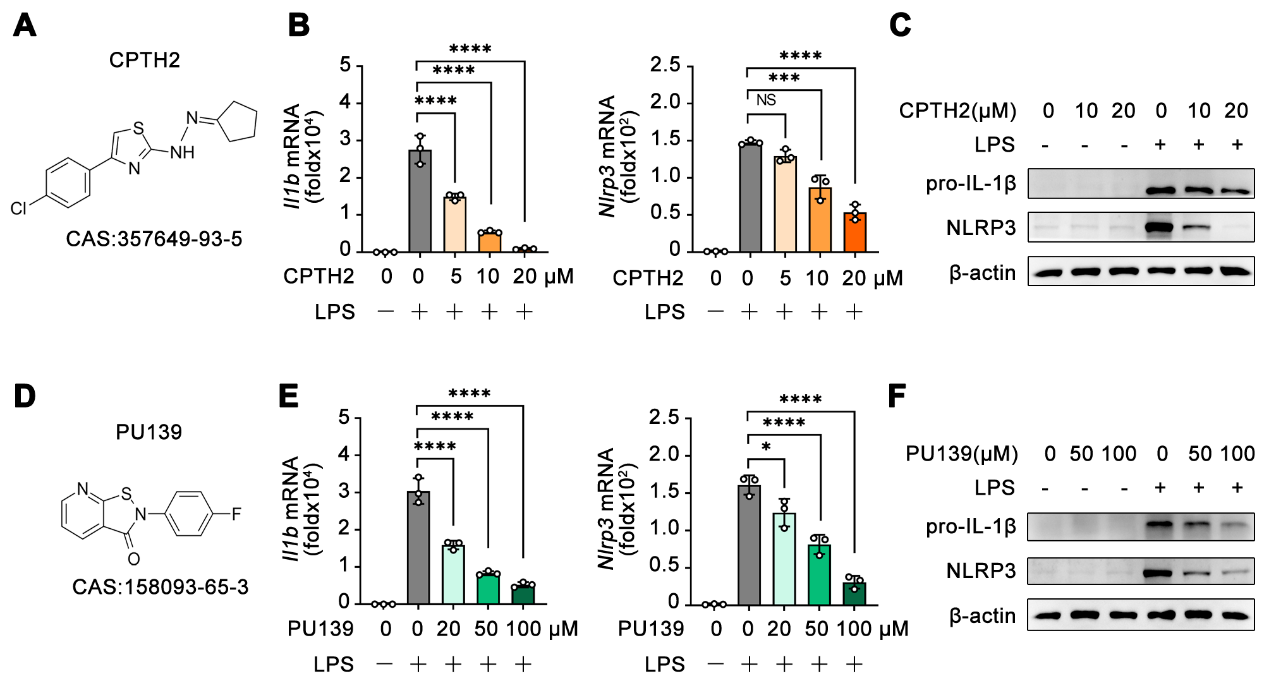
**

**Figure S5. CPTH2 and PU139 inhibit the priming of NLRP3 inflammasome.**

(**A**) Chemical formula and CAS information of CPTH2. (**B**-**C**) Q-PCR analysis of *Il1b* and *Nlrp3* mRNA levels (**B**) and immunoblot analysis of these proteins (**C**) in BMDMs pre-treated with the indicated amounts of CPTH2 followed by stimulation with LPS for 4 h. (**D**) Chemical formula and CAS information of PU139. (**E**-**F**) Q-PCR analysis of *Il1b* and *Nlrp3* mRNA levels (**E**) and immunoblot analysis of these proteins (**F**) in BMDMs pre-treated with the indicated amounts of PU139 followed by stimulation with LPS for 4 h. ^*^*P* < 0.05; ^***^*P* < 0.001; ^****^*P* < 0.001. One-way ANOVA (**B**, **E**).


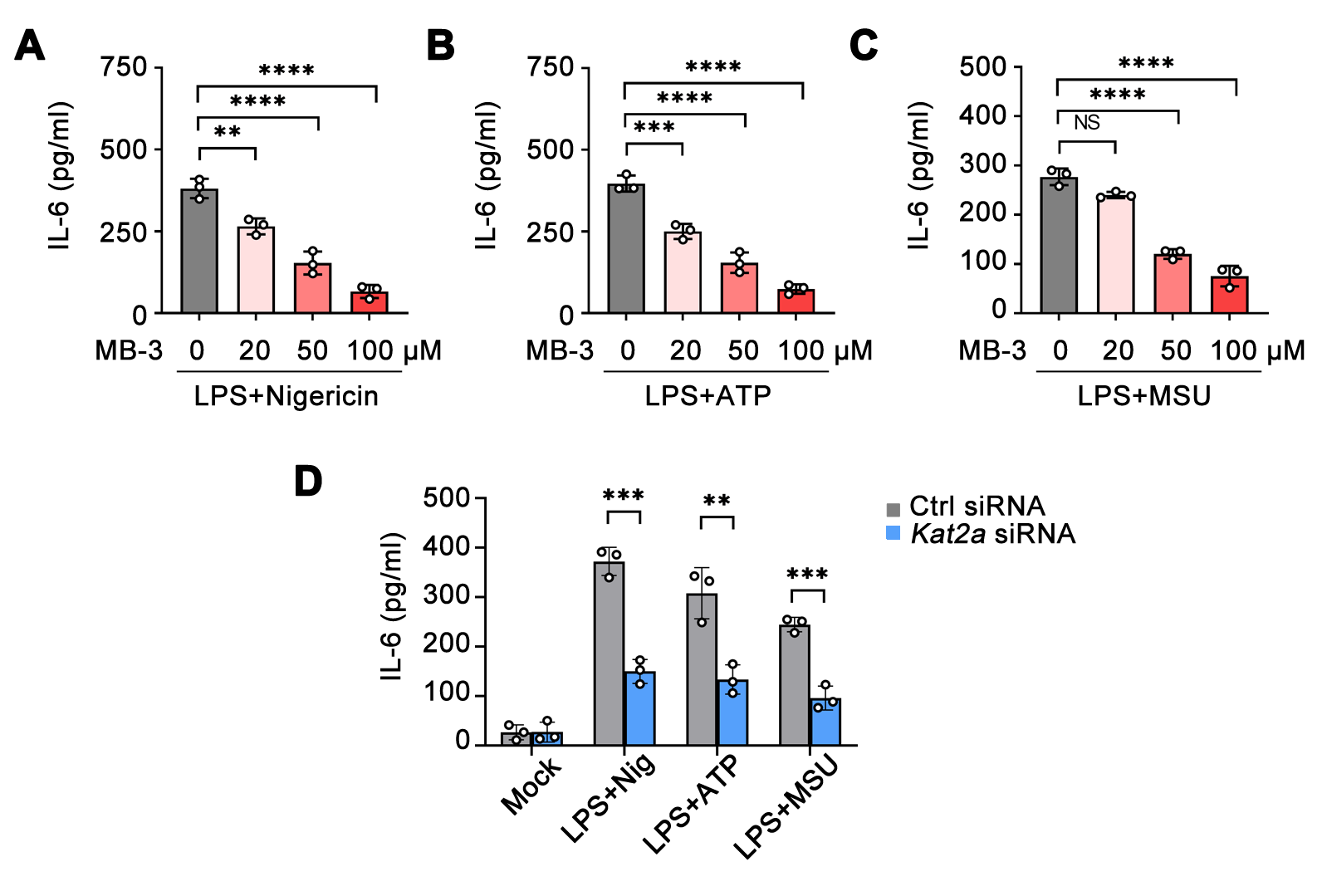


**Figure S6. KAT2A promotes IL-6 secretion during NLRP3 inflammasome activation.**

(**A**-**C**) ELISA analysis of IL-6 concentration in supernatants of LPS-primed mouse BMDMs pre-treated with MB-3 followed by stimulation with Nigericin (**A**), ATP (**B**) or MSU (**C**). (**D**) ELISA analysis of IL-6 concentration in supernatants of BMDMs transfected with control siRNA or *Kat2a* siRNA followed by treatment as indicated. ^**^*P* < 0.01; ^***^*P* < 0.001; ^****^*P* < 0.001. One-way ANOVA (**A**-**C**), unpaired Student's t test (**D**).





**Figure S7. MB-3 treatment inhibits the activation of NLRP3 inflammasome in arthritis mice.**

Immunoblot analysis of the indicated proteins in synovial tissues from CIA model mice administrated with DMSO or MB-3 (10 mg/kg body weight).


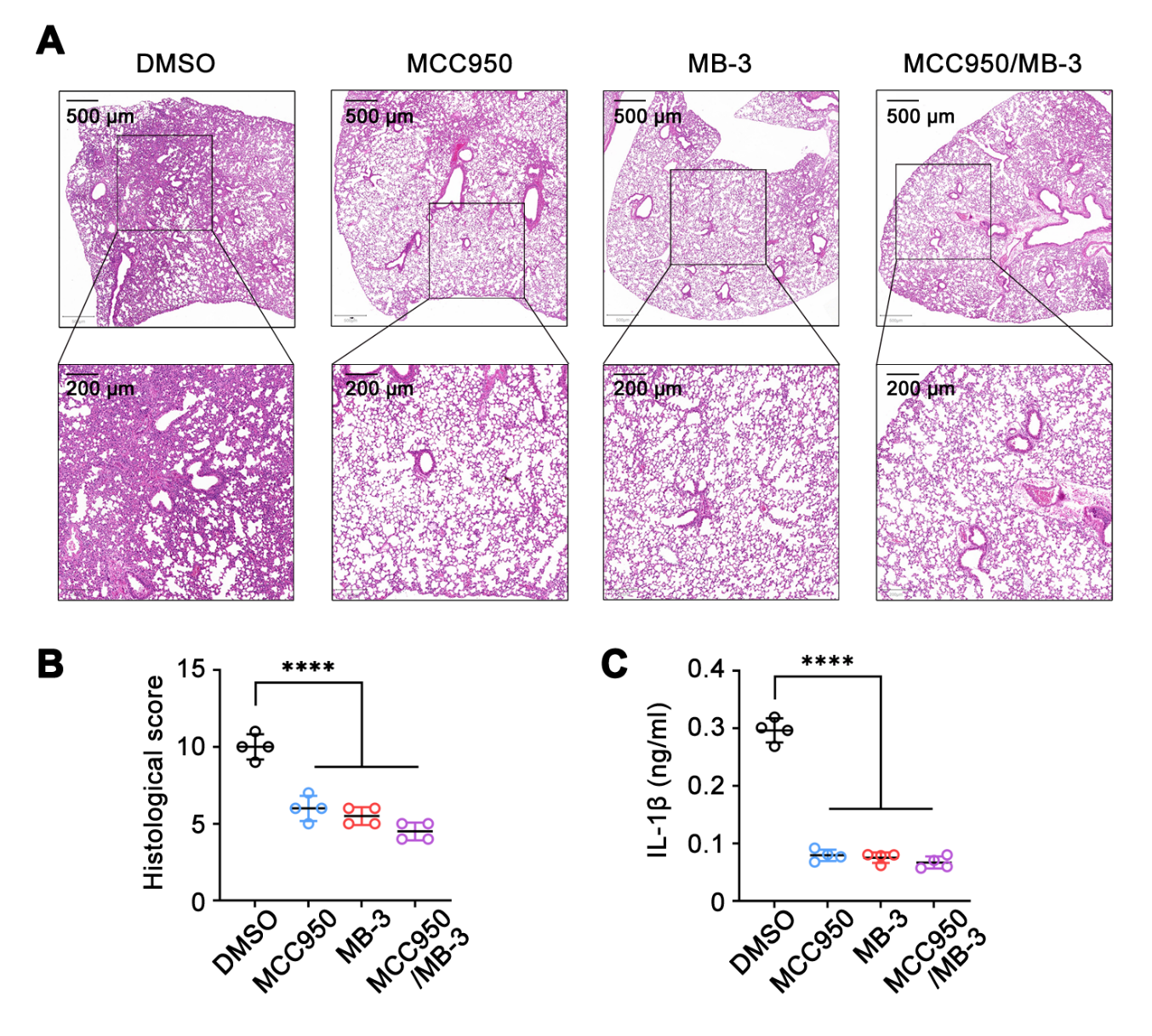


**Figure S8. The therapeutic effect of MB-3 is largely dependent on NLRP3 inflammasome.**

(**A-B**) H&E staining (**A**) and histological scores (**B**) of the lung tissues from the wild type mice pre-treated with MCC950, MB-3 or combined MB-3 and MCC950 followed by the intraperitoneal injection with LPS (12 mg/kg body weight) (n = 4 mice per group). Scale bar: 500 μm (upper) or 200 μm (lower). **(C)** ELISA analysis of IL-1β concentration in the sera of mice as in **A** (n = 4 mice per group). ^****^*P* < 0.001. One-way ANOVA (**B-C**).


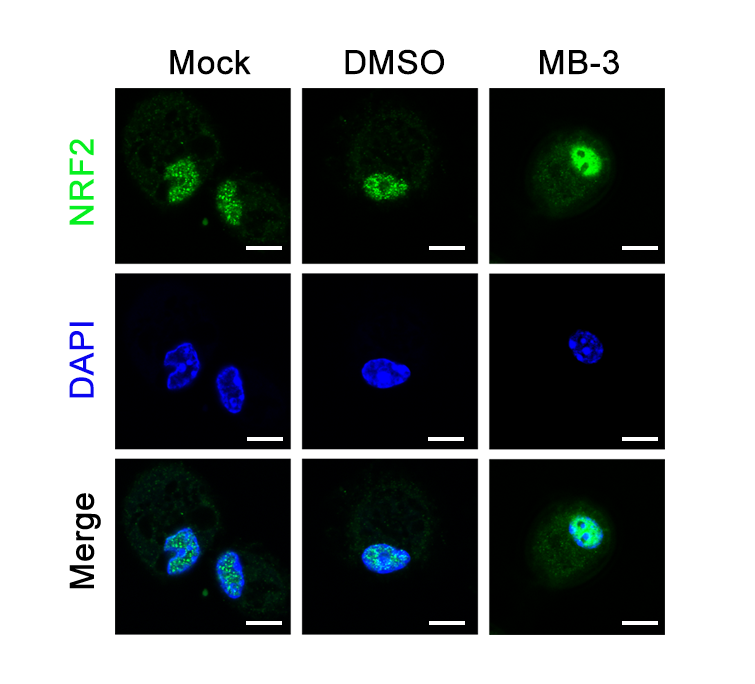


**Figure S9. MB-3 treatment promotes NRF2 expression in macrophages.**

Immunofluorescence analysis of NRF2 expression in BMDMs pre-treated with MB-3 (50 μM) or DMSO followed by stimulation with LPS (100 ng/ml) for 4 h. Scale bar: 10 μm.


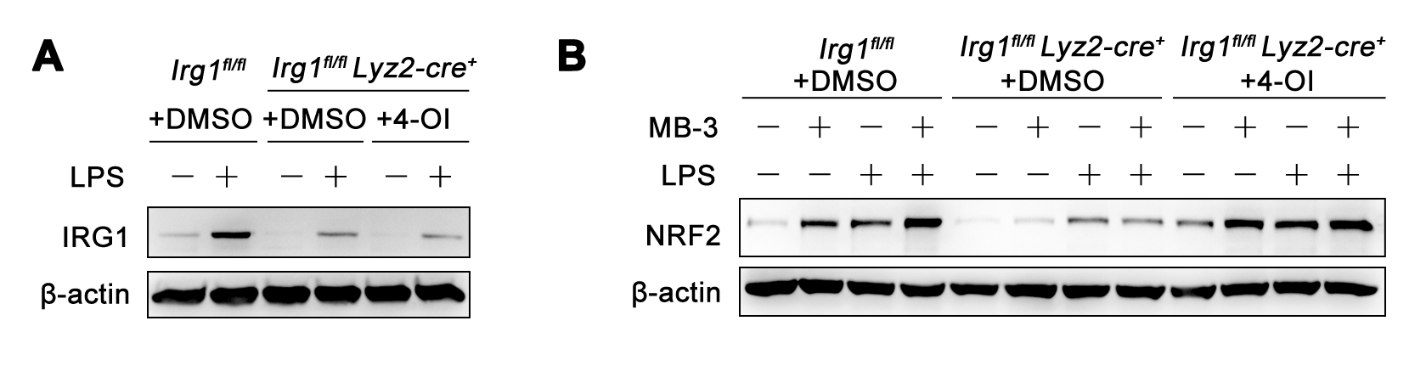


**Figure S10. IRG1/Itaconate axis is essential for the up-regulation of NRF2 mediated by MB-3.**

(**A**) Immunoblot analysis of IRG1 in BMDMs from WT and macrophage-conditional IRG1-deficient mice pre-treated with or without 4-OI (125 μM) followed by stimulation with LPS (100 ng/ml) for 4 h. (**B**) Immunoblot analysis of NRF2 in WT and IRG1-deficient BMDMs pre-treated with MB-3 (50 μM) followed by stimulation with LPS (100 ng/ml) for 4 h with or without 4-OI (125 μM).


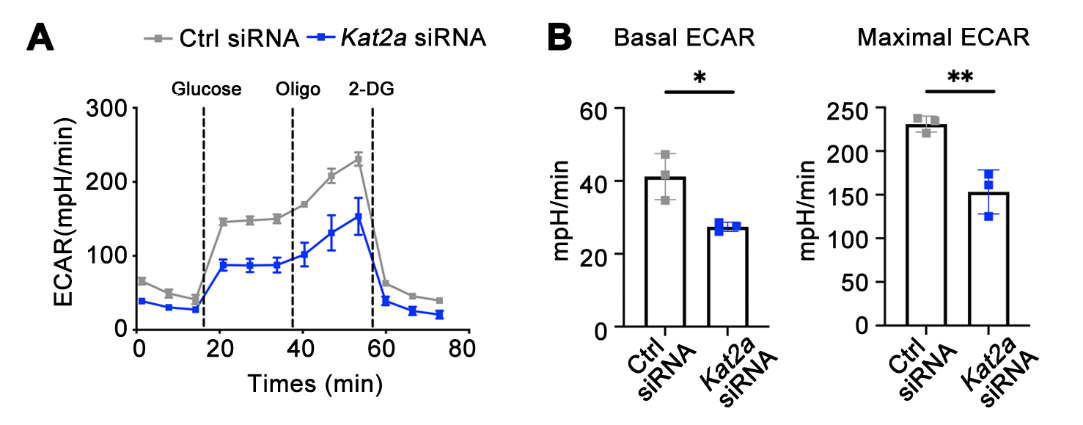


**Figure S11. KAT2A silencing inhibits glycolysis in macrophages.**

(**A-B**) ECAR analysis (**A**), basal ECAR and maximal ECAR (**B**) of BMDMs transfected with control siRNA or *Kat2a* siRNA followed by stimulation with LPS (100 ng/ml) for 4 h. ^*^*P* < 0.05; ^**^*P* < 0.01. Unpaired Student's t test (**B**).

**Table S1. Primers for Q-PCR analysis of mouse gene mRNA levels.**

| **Primers Sequences (5'-3')** |
| --- |
| *Kat2a* forward AACCTGAGCGAGTTGTGCC  *Kat2a* reverse GCCGGTTAATCTCGTCCTCTG  *Nlrp3* forward TGGATGGGTTTGCTGGGA  *Nlrp3* reverse CTGCGTGTAGCGACTGTT  *Il1b* forward GGTGTGTGACGTTCCCATTAGAC  *Il1b* reverse CATGGAGAATATCACTTGTTGGTTGA  *Il6* forward CACAGAGGATACCACTCCCAACA  *Il6* reverse TCCACGATTTCCCAGAGAACA  *Tnf* forward CATCTTCTCAAAATTCGAGTGACAA  *Tnf* reverse CCAGCTGCTCCTCCACTTG  *Nfe2l2* forward TCTTGGAGTAAGTCGAGAAGTGT  *Nfe2l2* reverse GTTGAAACTGAGCGAAAAAGGC  *Actb* forward AGGTCGGTGTGAACGGATTTG  *Actb* reverse TGTAGACCATGTAGTTGAGGTCA |

**Table S2. Primers for Q-PCR analysis of human gene mRNA levels.**

| **Primers Sequences (5'-3')** |
| --- |
| *KAT2A* forward GCAAGGCCAATGAAACCTGTA  *KAT2A* reverse TCCAAGTGGGATACGTGGTCA  *IL1B* forward ATGATGGCTTATTACAGTGGCAA  *IL1B* reverse GTCGGAGATTCGTAGCTGGA  *NLRP3* forward CGTGAGTCCCATTAAGATGGAGT  *NLRP3* reverse CCCGACAGTGGATATAGAACAGA  *ACTB* forward AGGTCGGTGTGAACGGATTTG  *ACTB* reverse CTCCTTAATGTCACGCACGAT |

**Table S3. Primers for Q-PCR analysis in ChIP assays.**

| **Primers** **Sequences (5'-3')** |
| --- |
| *Il1b* promoter forward TGATGATGTTGGCAAAGGAA  *Il1b* promoter reverse AAAAGCTAGAGTGCCCGTCA  *Nlrp3* promoter forward CGGGAAGCAATCTCTTCTGC  *Nlrp3* promoter reverse CGTGTCTGACCAGAACCCAT  *Nqo1* promoter forward GCCTACATAATCAGCCTGTG  *Nqo1* promoter reverse TTTGAGCCCATCCGTTTT |
